# SCOTfluors: Small, Conjugatable, Orthogonal, and Tunable Fluorophores for In Vivo Imaging of Cell Metabolism

**DOI:** 10.1002/anie.201900465

**Published:** 2019-04-12

**Authors:** Sam Benson, Antonio Fernandez, Nicole D. Barth, Fabio de Moliner, Mathew H. Horrocks, C. Simon Herrington, Jose Luis Abad, Antonio Delgado, Lisa Kelly, Ziyuan Chang, Yi Feng, Miyako Nishiura, Yuichiro Hori, Kazuya Kikuchi, Marc Vendrell

**Affiliations:** ^1^ Centre for Inflammation Research The University of Edinburgh EH16 4TJ Edinburgh UK; ^2^ UK Dementia Research Institute and EaStCHEM School of Chemistry The University of Edinburgh EH9 3FJ Edinburgh UK; ^3^ Edinburgh Cancer Research Centre EH4 2XR Edinburgh UK; ^4^ Research Unit on Bioactive Molecules Institute for Advanced Chemistry of Catalonia 08034 Barcelona Spain; ^5^ University of Barcelona Faculty of Pharmacy, Unit of Pharmaceutical Chemistry (CSIC Associated Unit) Barcelona Spain; ^6^ Graduate School of Engineering Osaka University Suita Japan

**Keywords:** cancer, fluorescence, imaging agents, metabolites, super-resolution imaging

## Abstract

The transport and trafficking of metabolites are critical for the correct functioning of live cells. However, in situ metabolic imaging studies are hampered by the lack of fluorescent chemical structures that allow direct monitoring of small metabolites under physiological conditions with high spatial and temporal resolution. Herein, we describe SCOTfluors as novel small‐sized multi‐colored fluorophores for real‐time tracking of essential metabolites in live cells and in vivo and for the acquisition of metabolic profiles from human cancer cells of variable origin.

Metabolites are essential biochemical components, with their transport and localization regulating most biological functions. Despite advances in fluorescence imaging to label biomolecules,^1^ there are few approaches to image small metabolites in live cells and intact organisms. Most metabolites do not contain groups that allow direct visualization and need to be modified with exogenous chromophores. However, fluorescent labels, in particular red and near‐infrared (NIR) fluorophores, are bulky structures that can impair metabolite traffic within cells. Our group has recently developed fluorogenic amino acids to label peptides without affecting their properties.^2^ Herein, we describe a new strategy for direct imaging of essential metabolites in live cells and in vivo using small‐sized multi‐color fluorophores.

Since the report by Ghosh and Whitehouse,^3^ nitrobenzodioxazole (NBD) has been widely used because of its small size and neutral character. These properties have facilitated labeling biomolecules with retention of their native properties.^4^ However, NBD (λ_em_≈540 nm) is incompatible with other green fluorescent reporters (e.g., GFP) and has limited application for in vivo use. To address these shortcomings, herein we report a collection of fluorophores, named SCOTfluors, with tunable emission covering the entire visible spectrum. SCOTfluors include the smallest fluorophores emitting in the NIR window (650–900 nm) reported to date (Figure 1).

**Figure 1:**
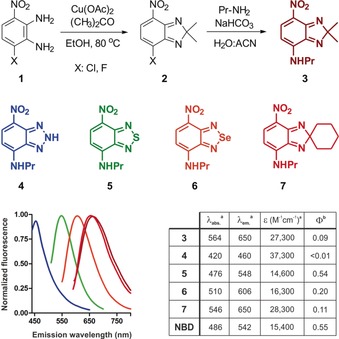
General synthetic procedure, structures, and spectral properties of SCOTfluors. a) Values determined in EtOH. b) QY in dioxane using acridine orange, fluorescein, rhodamine 101, and Cy5 as standards.

Several strategies have been described to optimize the optical properties of fluorophores for live‐cell imaging.^5^ For instance, the replacement of oxygen atoms with geminal dimethyl groups in rhodamine and fluorescein produced red‐shifted fluorophores with enhanced properties for bioimaging.^6^ We envisioned that the synthesis of nitrobenzodiazoles with different groups bridging the nitroaminoaniline core would render multi‐color fluorophores with tunable emission and enhanced capabilities for metabolite imaging in live cells. The preparation of SCOTfluors was achieved in two synthetic steps from the common intermediate **1** (Figure 1). First, the aminoaniline core was cyclized with different bridging groups. All these reactions proceeded similarly for fluoride and chloride derivatives (full list of analogues in the Supporting Information). Second, halogenated compounds (**2**, Figure 1) underwent substitution with primary and secondary amines to render the final fluorophores (**3**–**7**, Figure 1). Triazole derivatives (**4**) were synthesized by reaction with sodium nitrite in acidic media at r.t., thioderivatives (**5**) were obtained by condensation with *N*‐thionylaniline under heating, and selenium analogues (**6**) were prepared by reaction with SeO_2_ under reflux in EtOH. Finally, carbon derivatives (**3** and **7**) were synthesized by Cu‐catalyzed coupling using linear and cyclic ketones, respectively.

We examined the optical properties of SCOTfluors and compared them to the original NBD (Figure 1 and Figure S1). With the exception of triazoles (**4**), all compounds showed longer emission wavelengths than NBD, long Stokes shifts (around 80–100 nm), solvatochromic properties (Figure S2), and good photostability (Figure S3). Among SCOTfluors, Se‐ and C‐bridged derivatives display red and NIR emission, respectively, likely owing to reduced HOMO–LUMO gaps that result in bathochromic shifts in fluorescence emission, as with heteroatom‐bridged rhodamine and rhodol fluorophores.6b,6c, 7 To the best of our knowledge, this is the first example of C‐bridged nitrobenzodiazoles as fluorophores with NIR emission. Furthermore, C‐bridged derivatives are readily accessible through one‐step coupling of aminoaniline **1** with different ketones, representing a new platform for the direct synthesis of small NIR fluorophores. SCOTfluors proved compatible for experiments in live cells, showing no significant cytotoxicity in HeLa cells (Figure S4).

Then, we examined the properties of SCOTfluors for imaging the trafficking of essential metabolites under physiological conditions. Sphingolipids are critical components of membranes in the regulation of cellular metabolism. The dysregulation of sphingolipid metabolism is associated with several diseases (e.g., Gaucher and Niemann–Pick^8^) and its intracellular localization is crucial to understand metabolic disruption. We used the C‐bridged nitrobenzodiazole core to generate the NIR ceramide **8** (Figure 2 A) and monitor its intracellular localization over time by co‐staining with endoplasmic reticulum (ER) and lysosome markers. Spectral analysis confirmed that the optical properties of **8** were independent of the sphingoid base and therefore could be applicable to several types of biolipids (Figure 2 C and Figure S5). Compound **8** showed insignificant aggregation in water (Figure S6), and the incubation with liposomes highlighted its fluorogenic behavior, with around 15‐fold increase in emission (Figure 2 B and Figure S7). We exploited this property to visualize the recycling of ceramide **8** in real time in human A549 cells using fluorescence confocal microscopy.

**Figure 2:**
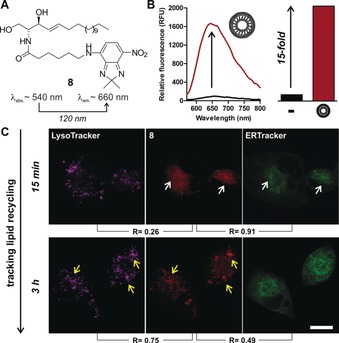
A) NIR‐fluorescent ceramide **8**. B) Emission of **8** in PBS (black) and in phosphatidylcholine/cholesterol (7:1) liposomes (red). C) Confocal microscopy images of A549 cells treated with **8** (50 μm, red), LysoTracker Blue (magenta) and ER Tracker Green (green) after 15 min (co‐localization=white arrows) and 3 h (co‐localization=yellow arrows). Total co‐localization coefficients (R) were determined using ImageJ. Scale bar=15 μm.

At short times (i.e., 15 min), the ceramide **8** was mainly found at the Golgi apparatus around the ER, as shown by high co‐localization with ER Tracker Green (*R*=91 %) but not LysoTracker Blue (*R*=26 %). Time‐lapse imaging demonstrated that the ceramide **8** translocated to the recycling lysosomes after 3 h (Figure 2 C) as highlighted by the increased co‐staining with LysoTracker Blue (*R*=75 %). Notably, these observations agree with prior reports of lipid mobilization,^9^ and could not be obtained with a fluorescein ceramide analog (Figure S8). These results confirm the suitability of our approach to prepare neutral NIR‐fluorescent probes to image biolipid function in cells.

We also examined whether SCOTfluors could be used to image in vivo tissues with high metabolic activity. Fluorescent deoxyglucose tracers can monitor glucose uptake in metabolically active cells and tissues,^10^ although few have been reported for in vivo use. We synthesized compound **9** (Figure 3 I) as an in vivo‐compatible glucose analog by conjugation of the nitrobenzoselenadiazole **6** with 2‐deoxyglucosamine. Notably, we performed the reaction with chloride and fluoride derivatives of **6** and observed increased reactivity and recovery for the latter (Figure S9). Compound **9** showed emission around 605 nm with a remarkable Stokes shift of 115 nm (Figure S10), enabling multiplexed imaging with blue and green fluorescent proteins (i.e., BFP and GFP, Figure 3 A–F). We examined the transport of **9** in HeLa cells transfected with EGFP‐tagged GLUT4, the main glucose transporter in mammalian cells. Fluorescence microscopy showed the uptake of **9** in GLUT4‐EGFP cells and co‐localization with the transporters (Figure 3 A–C). Notably, the uptake of **9** was blocked by competition with excess glucose (Figure 3 D–F) and was increased by pre‐treating HeLa cells with insulin^11^ (Figure 3 H and Figure S11). These results confirm that compound **9** is a functional substrate of GLUT4 and that enables dual tracking of glucose uptake and its transporters under physiological conditions. Finally, we tested compound **9** in vivo in zebrafish embryos to visualize regions of high glucose uptake. In vivo administration and imaging of compound **9** in wildtype zebrafish embryos indicated bright red fluorescence staining in regions of the developing brain (e.g., midbrain and hindbrain; Figure 3 J), which express GLUT2 transporters to supply glucose from circulation.^12^ We confirmed that the staining was dependent on the active transport of **9** through GLUT2 by examining *glut2* morpholino‐injected zebrafish, which have reduced levels of GLUT2. In vivo images of **9**‐treated *glut2* morpholino‐injected zebrafish showed much weaker fluorescence in the same regions (Figure 3 J). We also tested compound **6‐NEt_2_** as a control and observed no tissue‐specific staining in wildtype or in *glut2* morpholino‐injected zebrafish (Figure 3 J), highlighting the role of deoxyglucose to recognize GLUT2 transporters. Altogether, compound **9** can be used to image glucose uptake in vivo and to perform non‐invasive studies of glucose transport in whole organisms.

**Figure 3:**
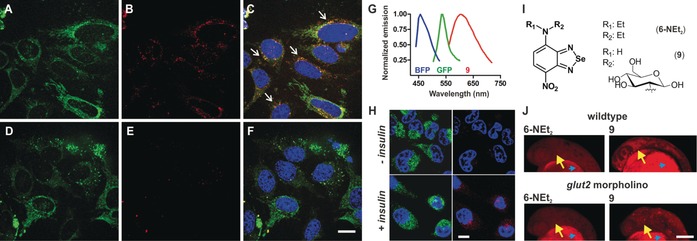
Fluorescence images of GLUT4‐EGFP HeLa cells treated with **9**. A–F) Green (GLUT4‐EGFP), red (**9**, 100 μm) and merged (Hoechst 33 342) images of HeLa cells without additional glucose (A–C) and in media containing 5 mm d‐glucose (D–F). White arrows identify co‐localization of GLUT4‐EGFP and **9**. Scale bar=10 μm. G) Fluorescence emission spectra of BFP (blue), GFP (green), and **9** (red). H) Insulin‐dependent (100 nm, 1 h) uptake of **9** (red, 100 μm) in GLUT4‐EGFP HeLa cells. I) Chemical structures of compounds **6‐NEt_2_** and **9**. J) In vivo images of the head in zebrafish embryos (28 h post fertilization, hpf) after injection of **6‐NEt_2_** or **9** (both 50 pmol) to the yolk sac (blue arrowheads). Fluorescence images were taken of wildtype zebrafish or zebrafish that had been injected at one cell stage with 4.2 ng anti‐sense *glut2* morpholino. Yellow arrows point at midbrain and hindbrain regions within the zebrafish embryo heads. Scale bar=100 μm.

Next, we used SCOTfluors to prepare the first red‐fluorescent analogue of lactic acid, an essential metabolite in muscle, blood, and cancer cells. Lactic acid is known as a carbon source in cancer cells and its uptake in tumours has been recently linked to aggressive oncological behaviour,^13^ yet little is known about its traffic and diffusion inside cancer cells. We conjugated the nitrobenzoselenadiazole **6** with l‐isoserine to produce compound **10** (Figure 4 A, *λ*_em_≈605 nm) as a probe to study the transport of lactic acid in live cells. First, we confirmed increased uptake in hypoxic (1 % O_2_) versus normoxic (20 % O_2_) cells, since lactic acid can accumulate in environments with low concentrations of oxygen (Figure 4 B).^14^

**Figure 4:**
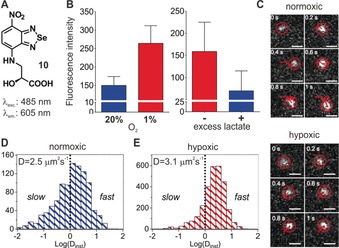
A) Structure of **10**. B) Fluorescence emission of live cells after incubation with **10** (100 μm) at different oxygen tensions (normoxia=blue; hypoxia=red) and in normoxic cells after competition with lactic acid (no lactate=red; 5 mm lactate=blue). Values as means and s.e.m. as error bars. C) TIRF tracking of fluorescent particles in untreated (top) and DMOG‐treated (10 μm, bottom) HeLa cells after incubation with compound **10** (100 μm) (Movies 1–2). Scale bars=1 μm. D) Histograms of the diffusion coefficients of fluorescent particles in normoxic (blue) and hypoxic (red) cells. Dotted lines delineate fast and slow diffusion species. Mean diffusion coefficients (*D*) were determined after averaging the tracks of multiple particles for each condition (*n*=1009 for untreated; *n*=2906 for DMOG‐treated).

We performed flow cytometry analysis to observe that hypoxic cells were significantly brighter than normoxic cells after incubation with the same concentration of compound **10**. We also performed competition assays between **10** and excess of lactic acid in normoxic cells, which markedly reduced the fluorescence staining, suggesting a common transporter for compound **10** and lactic acid in live cells (Figure 4 B). Encouraged by these results, we used total internal reflection fluorescence (TIRF) microscopy to image the real‐time diffusion of lactic acid in normoxic and hypoxic cancer cells with super‐resolution. For these studies, we used HeLa cells that had been treated or not with dimethyloxalylglycine (DMOG), a permeable prolyl 4‐hydroxylase inhibitor that upregulates hypoxia‐inducible factors.

We tracked the paths of over 1000 individual particles in both untreated (i.e., normoxic) and DMOG‐treated (i.e., hypoxic) cells after incubation with compound **10** and measured their respective intracellular diffusion coefficients (Figures 4 D‐E and Figure S12, Movies 1–4). Remarkably, particles in hypoxic cells showed higher mean diffusion coefficients than in normoxic cells, as well as a reduction of the slow diffusion species. Altogether, these results suggest that hypoxic tumors might display faster recycling rates for intracellular lactic acid than normoxic tumors and demonstrate the utility of compound **10** as a new probe for imaging lactic acid metabolism in live cells with high spatiotemporal resolution.

Finally, given the multi‐color capabilities and high stability of SCOTfluors under physiological and oxidative environments (Figure S13), we employed them to analyze the metabolic profiles of human cells from different origin. The groups of Chang and Rotello previously reported the discrimination of cancer cells using fluorescent dyes^15^ or host–guest arrays.^16^ In this study, we incubated human cancer cell lines with compounds **8**, **10**, and **11** (Figure 5) as respective analogues of ceramide, lactic acid, and glucose in order to obtain metabolic uptake signatures. First, we plated the cells at similar densities and incubated them with the probes under the same conditions. Next, we measured their fluorescence emission in the NIR, red, and green regions to determine their intracellular levels of ceramide, lactic acid, and glucose, respectively. Notably, different cancer cells presented variability in their metabolic uptake, as represented by their intracellular glucose/lactate and ceramide/lactate ratios (Figure 5). Whereas the biological implications of these results remain to be defined, these results demonstrate that SCOTfluors can generate multiplexed metabolic readouts from live cells, which is not possible in other imaging modalities.

**Figure 5:**
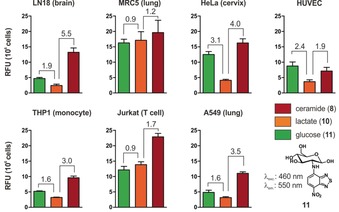
Multi‐color metabolic profiles of human cancer cells. Relative fluorescence intensities of **8**, **10**, and **11** were measured in different human cell lines. Emission wavelengths: **8** (650 nm), **10** (610 nm), **11** (550 nm). Values are presented as fluorescence emission per 10^6^ cells after normalization against the fluorescence intensity of the respective solutions in aqueous buffer. Fluorescence fold ratios glucose vs. lactate (left) and ceramide vs. lactate (right) are calculated for cell type. Data presented as means and s.e.m. as error bars.

In conclusion, we developed SCOTfluors as small‐sized fluorophores covering the entire visible spectrum. SCOTfluors are readily obtained by bridging aminoanilines with different groups and include the smallest NIR‐emitting fluorophores to date. We validated SCOTfluors for real‐time and in situ imaging of different small metabolites (e.g., lipids and sugars) in live cells and in vivo, as well as their combination to generate multi‐color fingerprints in cells. The tunability and versatility of SCOTfluors will enable non‐invasive bioimaging studies of essential metabolites that cannot be performed with conventional fluorophores.

## Conflict of interest

The University of Edinburgh has filed an invention disclosure form to protect part of the technology described in the study.

## Supporting information

As a service to our authors and readers, this journal provides supporting information supplied by the authors. Such materials are peer reviewed and may be re‐organized for online delivery, but are not copy‐edited or typeset. Technical support issues arising from supporting information (other than missing files) should be addressed to the authors.

Supplementary

Supplementary

Supplementary

Supplementary

Supplementary
