# Supplementary material for: SCOTfluors: Small, Conjugatable, Orthogonal, and Tunable Fluorophores for In Vivo Imaging of Cell Metabolism
Source: Angew Chem Int Ed Engl. 2019 Apr 12;58(21):6911–5. doi: 10.1002/anie.201900465 (PMC6563150; doi:10.1002/anie.201900465)
Supplement: Supplementary file 1 — Supplementary [file ANIE-58-6911-s001.pdf]

Angewandte Chemie
Eine Zeitschrift der Gesellschaft Deutscher Chemiker

Supporting Information

SCOTfluors: Small, Conjugatable, Orthogonal, and Tunable Fluorophores for In Vivo Imaging of Cell Metabolism

*Sam Benson[+], Antonio Fernandez[+], Nicole D. Barth, Fabio de Moliner, Mathew H. Horrocks, C. Simon Herrington, Jose Luis Abad, Antonio Delgado, Lisa Kelly, Ziyuan Chang, Yi Feng, Miyako Nishiura, Yuichiro Hori, Kazuya Kikuchi, and Marc Vendrell**

anie_201900465_sm_miscellaneous_information.pdf

Electronic Supporting Information

Table of Contents

1. Chemical Synthesis.

2. NMR Spectra.

3. Supplementary Figures.

4. Biological Assays.

5. Supplementary Movie Legends.

6. Supplementary References.

1. Chemical Synthesis.

General materials.

Commercially available reagents were used without further purification. Thin-layer chromatography was conducted on Merck silica gel 60 F254 sheets and visualized by UV (254 and 365 nm). Silica gel (particle size 35−70 μm) was used for column chromatography. ^{1}H and ^{13}C NMR spectra were recorded in a Bruker Avance 500 spectrometer (at 500 and 126 MHz, respectively). Data for ^{1}H NMR spectra are reported as chemical shift δ (ppm), multiplicity, coupling constant (Hz), and integration. Data for ^{13}C NMR spectra are reported as chemical shifts relative to the solvent peak. HPLC−MS analysis was performed on a Waters Alliance 2695 separation module connected to a Waters PDA2996 photo-diode array detector and a ZQ Micromass mass spectrometer (ESI-MS) with a Phenomenex column (C_{18}, 5 μm, 4.6 × 150 mm). Conjugates were purified using a Waters semipreparative HPLC system using a Phenomenex column (C_{18} Axial, 10 μm, 21.2 × 150 mm) and UV detection.

Synthesis of 3-chloro and 3-fluoro-6-nitrobenzene-1,2-diamine (1-Cl, 1-F).

Nitrobenzoselenadiazoles (2.9 mmol) were dissolved in HCl_{conc} (30 mL) and HI 57% (7.5 mL) was added dropwise. The reaction was stirred for 1 h at r.t. and then a saturated aqueous solution of Na_2SO_3 (100 mL) was added. Afterwards, an aqueous solution of 2 N NaOH was added dropwise until the pH reached 8. The solution was filtered through Celite and extracted with EtOAc (4 × 100 mL). The organic phase was dried over anhydrous $MgSO_4$ and the solvent was removed under reduced pressure to render dark red solids. Crude product were purified by column chromatography (EtOAc:Hexane 3:7).

3-chloro-6-nitrobenzene-1,2-diamine (1-Cl) (77% yield, red solid) ^{1}H NMR (500 MHz, DMSO-d_6) δ 7.34 (d, J = 9.4 Hz, 1H), 7.27 (s, 2H), 6.62 (d, J = 9.4 Hz, 1H), 5.50 (s, 2H). ^{13}C NMR (126 MHz, DMSO-d_6) δ 136.1, 133.9, 130.0, 120.9, 116.5, 113.6. m/z (ESI): calcd for $C_6H_6ClN_3$[M]: 187.0, found: 187.4.

3-fluoro-6-nitrobenzene-1,2-diamine (1-F) (85% yield, orange solid) ^{1}H NMR (500 MHz, DMSO-d_6) δ 7.40 (dd, J = 9.6, 5.8 Hz, 1H), 7.14 (s, 2H), 6.52 (t, J = 9.7 Hz, 1H), 5.15 (s, 2H). ^{13}C NMR (126 MHz, DMSO-d_6) δ 153.6, 151.7, 137.8, 128.6, 124.3 (d, J_{C-F} = 16.2 Hz), 114.2 (d, J_{C-F} = 10.1 Hz), 104.8 (d, J_{C-F} = 23.3 Hz). m/z (ESI): calcd for $C_6H_7FN_3O_2^+$ [M+H]$^+$: 172.0, found: 172.5.

Synthesis of nitrobenzotriazoles (4-Cl, 4-F).

To a solution of **1-Cl** or **1-F** (0.16 mmol) in H_2O: AcOH (10:1, 2 mL) was added $NaNO_2$ (0.22 mmol) and the reaction was stirred for 30 min at r.t. Then, the mixture was extracted with EtOAc (2 × 30 mL), washed with 2 M HCl (10 mL), $NaHCO_{3(aq)}$ (10 mL) and brine (10 mL), dried over $MgSO_4$ and the solvent was removed under reduced pressure to give a light brown solid, which was purified by column chromatography (EtOAc:Hexane 6:4).

4-chloro-7-nitro-2H-benzo[d][1,2,3]triazole (4-Cl) (98% yield, yellow solid) ^{1}H NMR (500 MHz, DMSO-d_6) δ 8.45 (d, J = 8.4 Hz, 1H), 7.75 (d, J = 8.4 Hz, 1H). ^{13}C NMR (126 MHz, DMSO-d_6) δ 144.6, 133.0, 131.9, 128.2, 125.5, 124.1. m/z (ESI): calcd for $C_6H_4ClN_4O_2^+$ [M+H]$^+$: 199.0, found: 199.6.

4-fluoro-7-nitro-2H-benzo[d][1,2,3]triazole (4-F) (66% yield, yellow solid) ^{1}H NMR (500 MHz, DMSO-d_6) δ 8.55 (dd, J = 8.7, 3.9 Hz, 1H), 7.50 (dd, J= 9.7, 8.7 Hz, 1H). ^{13}C NMR (126 MHz, DMSO-d_6) δ 158.0, 155.9, 136.6, 130.6, 127.3 (d, J_{C-F} = 9.7 Hz), 109.7 (d, J_{C-F} = 20.1 Hz). m/z (ESI): calcd for $C_6H_4FN_4O_2^+$ [M+H]$^+$: 183.0, found: 183.5.

Synthesis of benzothiadiazoles (5-Cl, 5-F).

To a solution of **1-Cl** or **1-F** (0.7 mmol) in toluene (1 mL), PhNSO (5.0 mmol) was added and the reaction was heated at 120 °C for 5 h. Then, the solvent was removed under reduced pressure and the crude was purified by column chromatography (EtOAc:Hexane 4:6).

4-chloro-7-nitrobenzo[c][1,2,5]thiadiazole (5-Cl) (65% yield, yellow solid) ^{1}H NMR (500 MHz, DMSO-*d*$_6$) δ 8.69 (d, *J* = 8.2 Hz, 1H), 8.12 (d, *J* = 8.2 Hz, 1H). ^{13}C NMR (126 MHz, DMSO-*d*$_6$) δ 153.2, 146.6, 138.7, 132.4, 128.8, 128.1. m/z (ESI): calcd for $C_6H_2ClN_3O_2S^+$ [M]$^+$: 214.9, found: 214.9.

4-fluoro-7-nitrobenzo[c][1,2,5]thiadiazole (5-F) (68% yield, yellow solid) ^{1}H NMR (500 MHz, DMSO-*d*$_6$) δ 8.80 (dd, *J* = 8.5, 4.5 Hz, 1H), 7.79 (dd, *J* = 9.4, 8.6 Hz, 1H). ^{13}C NMR (126 MHz, DMSO-*d*$_6$) δ 157.3, 155.1, 148.4 (d, $J_{C\text{-}F}$ = 3.6 Hz), 146.3 (d, $J_{C\text{-}F}$ = 15.7 Hz), 136.5, 130.5 (d, $J_{C\text{-}F}$ = 9.9 Hz), 112.3 (d, $J_{C\text{-}F}$ = 19.5 Hz). m/z (ESI): calcd for $C_6H_2FN_3O_2S^+$ [M]$^+$: 199.0, found: 199.0.

Synthesis of 4-chloro and 4-fluorobenzoselenadiazoles.

SeO_2 (4.5 mmol) was added to a solution of 3-chlorobenzene-1,2-diamine or 3-fluorobenzene-1,2-diamine (3.7 mmol) in EtOH (20 mL) and the reaction was refluxed for 30 min. Then, the solvent was removed under reduced pressure and the crude products were purified by column chromatography (Hexane:EtOAc 7:3).

4-chlorobenzo[c][1,2,5]selenadiazole (91% yield, white solid) ^{1}H NMR (500 MHz, DMSO-*d*$_6$) δ 7.84 (dd, *J* = 9.0, 1.0 Hz, 1H), 7.70 (dd, *J* = 7.1, 0.9 Hz, 1H), 7.53 (dd, *J* = 9.0, 7.1 Hz, 1H). ^{13}C NMR (126 MHz, DMSO-*d*$_6$) δ 160.2, 156.8, 129.7, 128.5, 126.9, 123.0. m/z (ESI): calcd for $C_6H_4ClN_2Se^+$ [M+H]$^+$: 218.9, found: 218.9.

4-fluorobenzo[c][1,2,5]selenadiazole (90% yield, white solid) ^{1}H NMR (500 MHz, $CDCl_3$) δ 7.69 (ddd, *J* = 9.2, 0.8, 0.4 Hz, 1H), 7.47 (ddd, *J* = 9.1, 7.3, 5.2 Hz, 1H), 7.10 (ddd, *J* = 10.1, 7.3, 0.9 Hz, 1H). ^{13}C NMR (126 MHz, $CDCl_3$) δ 155.0, 145.2, 129.0, 123.4, 119.6 (d, *J* = 5.5 Hz), 111.0 (d, *J* = 17.0 Hz). m/z (ESI): calcd for $C_6H_4FN_2Se^+$ $[M+H]^+$: 202.9, found: 202.9.

General procedure for the synthesis of nitrobenzoselenadiazoles (6-Cl, 6-F).

4-Chloro or 4-fluorobenzoselenadiazole (2.3 mmol) was dissolved in H_2SO_4 (5 mL) and HNO_3 (1.5 mL) was added dropwise at 0 ^{o}C. After 15 min, the reaction was quenched by dropwise addition of H_2O (100 mL), leading to the formation of a yellow precipitate. The organic phase was extracted with EtOAc (3 × 150 mL) and dried over anhydrous $MgSO_4$. The solvent was removed under reduced pressure and the crude product was purified by column chromatography (Hexane:EtOAc 4:6).

4-chloro-7-nitrobenzo[c][1,2,5]selenadiazole (6-Cl) (97% yield, yellow solid) ^{1}H NMR (500 MHz, DMSO-d_6) δ 8.49 (d, *J* = 8.0 Hz, 1H), 7.93 (d, *J* = 8.0 Hz, 1H). ^{13}C NMR (126 MHz, DMSO-d_6) δ 157.2, 150.6, 140.3, 133.6, 127.8, 126.5. m/z (ESI): calcd for $C_6H_3ClN_3O_2Se^+$ $[M+H]^+$: 263.9, found: 263.9.

4-fluoro-7-nitrobenzo[c][1,2,5]selenadiazole (6-F) (89% yield, yellow solid) ^{1}H NMR (500 MHz, DMSO-d_6) δ 8.61 (dd, *J* = 8.4, 4.7 Hz, 1H), 7.55 (dd, *J* = 9.7, 8.4 Hz, 1H). ^{13}C NMR (126 MHz, DMSO-d_6) δ 157.7, 155.5, 152.3 (d, $J_{C\text{-}F}$ = 2.9 Hz), 133.2, 129.5 (d, $J_{C\text{-}F}$ = 10.0 Hz), 110.1 (d, $J_{C\text{-}F}$ = 20.4 Hz). m/z (ESI): calcd for $C_6H_3FN_3O_2Se^+$ $[M+H]^+$: 247.9, found: 247.7.

Synthesis of C-bridged benzoimidazoles (2-Cl, 2-F, 7-Cl, 7-F).

To a solution of **1-Cl** or **1-F** (0.3 mmol) in EtOH (5 mL), $Cu(OAc)_2$ (0.015 mmol) was added followed by the corresponding ketone (30 mmol). The resulting mixture was heated at 80 °C overnight. Then, the reaction mixture was filtered through Celite and the solvent was removed under reduced pressure to give crude products, which were purified by column chromatography (DCM:Hexane 1:1).

4-chloro-2,2-dimethyl-7-nitro-2H-benzo[d]imidazole (2-Cl) (82% yield, red solid) ^{1}H NMR (500 MHz, $CDCl_3$) δ 7.25 (d, J = 9.4 Hz, 1H), 6.46 (d, J = 9.4 Hz, 1H), 1.66 (s, 6H). ^{13}C NMR (126 MHz, $CDCl_3$) δ 138.6, 138.0, 132.2, 126.6, 117.9, 114.0, 80.9, 30.7. m/z (ESI): calcd for $C_9H_9ClN_3O_2^+$ $[M+H]^+$: 226.0, found: 226.5.

4-fluoro-2,2-dimethyl-7-nitro-2H-benzo[d]imidazole (2-F) (79% yield, red solid) ^{1}H NMR (500 MHz, $CDCl_3$) δ 7.33 (dd, J = 9.6, 4.6 Hz, 1H), 6.35 (dd, J = 9.6, 9.1 Hz, 1H), 1.64 (s, 6H). ^{13}C NMR (126 MHz, $CDCl_3$) δ 150.3, 148.3, 141.4 (d, $J_{C\text{-}F}$ = 11.3 Hz), 126.4 (d, $J_{C\text{-}F}$ = 17.0 Hz), 115.3 (d, $J_{C\text{-}F}$ = 8.4 Hz), 107.3 (d, $J_{C\text{-}F}$ = 22.1 Hz), 81.8, 30.6. m/z (ESI): calcd for $C_9H_9FN_3O_2^+$ $[M+H]^+$: 210.1, found: 209.8.

4-chloro-7-nitrospiro[benzo[d]imidazole-2,1'-cyclohexane] (7-Cl) (94% yield, red solid) ^{1}H NMR (500 MHz, Methanol-d_4) δ 7.08 (d, J = 9.4 Hz, 1H), 6.36 (d, J = 9.4 Hz, 1H), 1.89 – 1.81 (m, 4H), 1.73 (m, 4H), 1.64 – 1.56 (m, 2 H). ^{13}C NMR (126 MHz, Methanol-d_4) δ 139.4, 139.0, 125.4, 117.1, 112.6, 111.8, 82.6, 38.9, 24.4, 22.2. m/z (ESI): calcd for $C_{12}H_{13}ClN_3O_2^+$ $[M+H]^+$: 266.0, found: 266.4.

4-fluoro-7-nitrospiro[benzo[d]imidazole-2,1'-cyclohexane] (7-F) (80% yield, red solid) ^{1}H NMR (500 MHz, Methanol-d_4) δ 7.17 (dd, J = 9.6, 4.5 Hz, 1H), 6.30 (t, J = 9.5 Hz, 1H), 1.93 – 1.86 (m, 4H), 1.70 (ddt, J = 9.7, 6.4, 3.0 Hz, 4H), 1.66 – 1.57 (m, 2H). ^{13}C NMR (126 MHz, Methanol-d_4) δ 149.0, 147.1, 142.0 (d, $J_{C\text{-}F}$ = 11.9 Hz), 127.6 (d,

$J_{C\text{-}F}$ = 17.1 Hz), 124.4, 112.9 (d, $J_{C\text{-}F}$ = 8.4 Hz), 106.4 (d, $J_{C\text{-}F}$ = 22.9 Hz), 83.4, 38.8, 24.4, 22.3. m/z (ESI): calcd. for $C_{12}H_{12}FN_3O_2^+$ [M+H]$^+$: 250.1, found: 250.3.

General procedure for amine incorporation

To a solution of **4-F**, **5-F** or **6-F** (0.04 mmol) in MeCN (1 mL) were added the corresponding amine (0.06 mmol). The mixture was stirred at r.t. until TLC monitoring showed complete consumption of the starting material. Volatiles were removed under reduced pressure and the crude was purified by column chromatography (DCM:MeOH 98:2).

7-nitro-*N*-propyl-2H-benzo[d][1,2,3]triazol-4-amine (4) (17% yield, yellow solid) ^{1}H NMR (500 MHz, Methanol-*d4*) δ 8.24 (d, *J* = 9.0 Hz, 1H), 6.50 (d, *J* = 9.1 Hz, 1H), 3.75 – 3.43 (m, 2H), 1.88 – 1.72 (m, 2H), 1.08 (t, *J* = 7.4 Hz, 3H). ^{13}C NMR (126 MHz, Methanol-*d4*) δ 152.9, 140.3, 134.5, 128.3, 120.7, 100.4, 29.4, 22.0, 10.3. m/z (ESI): calcd for $C_9H_{12}N_5O_2^+$ [M+H]$^+$: 221.1, found: 221.3.

7-nitro-*N*-propylbenzo[c][1,2,5]thiadiazol-4-amine (5) (90% yield, orange solid) ^{1}H NMR (500 MHz, Methanol-*d4*) δ 8.67 (d, *J* = 9.0 Hz, 1H), 6.54 (d, *J* = 8.9 Hz, 1H), 3.51 (t, *J* = 7.2 Hz, 2H), 1.90 – 1.77 (m, 2H), 1.08 (t, *J* = 7.4 Hz, 3H). ^{13}C NMR (126 MHz, Methanol-*d4*) δ 148.4, 147.6, 146.7, 134.2, 126.7, 98.7, 44.6, 21.5, 10.3. m/z (ESI): calcd for $C_9H_{11}N_4O_2S^+$ [M+H]$^+$: 239.0, found: 238.8.

7-nitro-*N*-propylbenzo[c][1,2,5]selenadiazol-4-amine (6) (72% yield, red solid) ^{1}H NMR (500 MHz, DMSO-*d*6) δ 8.58 (d, *J* = 9.0 Hz, 1H), 6.39 (d, *J* = 9.0 Hz, 1H), 3.42 (q, *J* = 6.8 Hz, 2H), 1.80 – 1.63 (m, 2H), 0.95 (t, *J* = 7.4 Hz, 3H). ^{13}C NMR (126 MHz, DMSO) δ 152.6, 152.4, 149.1, 135.9, 128.4, 97.7, 45.0, 21.8, 11.8. m/z (ESI): calcd for $C_6H_{11}N_4O_2Se^+$ [M+H]$^+$: 287.0, found: 286.8.

***N,N*-diethyl-7-nitrobenzo[c][1,2,5]selenadiazol-4-amine (6-NEt$_2$)** (70% yield, red solid) ^{1}H NMR (500 MHz, DMSO-*d*$_6$) δ 8.50 (d, *J* = 9.4 Hz, 1H), 6.47 (d, *J* = 9.4 Hz, 1H), 4.01 (q, *J* = 7.0 Hz, 4H), 1.32 (t, *J* = 7.0 Hz, 6H). ^{13}C NMR (126 MHz, DMSO-*d*$_6$) δ 153.9, 152.4, 148.7, 134.1, 128.2, 101.9, 47.8, 13.2. m/z (ESI): calcd for $C_{10}H_{12}N_4O_2SeNa^+$ [M+Na]$^+$: 323.0, found: 323.1.

To a solution of **2-F** or **7-F** (0.05 mmol) in MeCN (1 mL), $NaHCO_3$ (0.13 mmol) in H_2O was added, followed by the amine (0.05 mmol) and the reaction was heated at 65 ^oC for 3h. The reaction mixture was acidified with 0.2 N HCl and extracted with EtOAc. The organic layer was dried over anhydrous $MgSO_4$, the solvent was removed under reduced pressure and the crude product was purified by column chromatography (DCM:MeOH 95:5).

2,2-dimethyl-7-nitro-*N*-propyl-2H-benzo[d]imidazol-4-amine (3) (41% yield, purple solid) ^{1}H NMR (500 MHz, Methanol-*d*$_4$) δ 8.52 (d, *J* = 10.0 Hz, 1H), 5.75 (d, *J* = 10.0 Hz, 1H), 2.76 (t, *J* = 7.3 Hz, 2H), 1.67 – 1.57 (m, 2H), 1.56 (s, 6H), 0.99 (t, *J* = 7.4 Hz, 3H). ^{13}C NMR (126 MHz, Methanol-d4) δ 180.7, 158.6, 154.3, 145.5, 110.2, 103.0, 42.0, 22.9, 21.9, 9.9. m/z (ESI): calcd for $C_{12}H_{17}N_4O_2^+$ [M+H]$^+$: 249.1, found: 249.2.

6-((2,2-dimethyl-7-nitro-2H-benzo[d]imidazol-4-yl)amino)hexanoic acid (3-Ahx) (40% yield, purple solid) ^{1}H NMR (500 MHz, Methanol-*d*$_4$) δ 8.53 (d, *J* = 8.9 Hz, 1H), 5.93 (d, *J* = 9.0 Hz, 1H), 3.49 (t, *J* = 7.2 Hz, 2H), 2.32 (t, *J* = 7.4 Hz, 2H), 1.78 (p, *J* = 7.4 Hz, 2H), 1.75 – 1.64 (m, 2H), 1.60 (s, 6H), 1.53 – 1.42 (m, 2H). ^{13}C NMR (126 MHz, Methanol-*d*$_4$) δ 176.9, 155.1, 151.6, 151.0, 145.0, 125.5, 105.6, 95.5, 43.1, 34.1, 27.7, 26.2, 24.6, 21.1. m/z (HRMS ESI): calcd for $C_{15}H_{21}N_4O_4^+$ [M+H]$^+$: 321.1561, found: 321.1557.

7-nitro-*N*-propylspiro[benzo[d]imidazole-2,1'-cyclohexan]-4-amine (7) (25% yield, purple solid) ^{1}H NMR (500 MHz, Methanol-d_4) δ 8.53 (d, *J* = 8.9 Hz, 1H), 5.95 (d, *J* = 9.0 Hz, 1H), 3.45 (t, *J* = 7.3 Hz, 2H), 2.07 – 1.90 (m, 4H), 1.78 (m, *J* = 7.4 Hz, 2H), 1.72 – 1.60 (m, 4H), 1.33 – 1.22 (m, 2H), 1.05 (t, *J* = 7.4 Hz, 3H). ^{13}C NMR (126 MHz, Methanol-d_4) δ 155.1, 151.4, 144.6, 125.6, 108.5, 95.6, 44.9, 32.9, 25.1, 24.2, 21.4, 10.3. m/z (ESI): calcd for $C_{15}H_{21}N_4O_2^+$ [M+H]$^+$: 289.1, found: 289.1.

Synthesis of lipid analog 8

To a solution of compound **3-Ahx** (5 mg, 0.016 mmol) in DMF:DCM (1:4, 0.5 mL), COMU (8.4 mg, 0.019 mmol) was added. After 5 min of stirring at r.t., sphingosine (6 mg, 0.019 mmol) dissolved in DMF:DCM (1:4, 0.4 mL) was added, followed by DIPEA (3.6 µL, 0.019 mmol). After stirring for 15 min at r.t. under N_2, the mixture was poured into H_2O:DCM (1:1, 8 mL) and the organic phase was washed with H_2O (2 mL), brine (2 x 2mL) and dried over anhydrous $MgSO_4$. The solvent was removed under reduced pressure, and the product was isolated after column chromatography (gradient from pure DCM to DCM:MeOH, 95:5).

***N*-((2S,3R,E)-1,3-dihydroxyoctadec-4-en-2-yl)-6-((2,2-dimethyl-7-nitro-2H-benzo[d]imidazol-4-yl)amino)hexanamide** (27% yield, purple solid) ^{1}H NMR (500 MHz, Methanol-d_4) δ 8.53 (d, *J* = 8.9 Hz, 1H), 5.92 (d, *J* = 8.9 Hz, 1H), 5.77 – 5.64 (m, 1H), 5.52 – 5.41 (m, 1H), 4.38 (q, *J* = 7.1 Hz, 1H), 3.80 – 3.64 (m, 2H), 3.57 – 3.50 (m, 2H), 2.26 (td, *J* = 7.3, 1.8 Hz, 2H), 2.14 – 2.00 (m, 4H), 1.83 – 1.64 (m, 4H), 1.60 (s, 6H), 1.43 – 1.34 (m, 2H), 1.31 (s, 22H), 0.92 (t, *J* = 6.9 Hz, 3H). ^{13}C NMR (126 MHz, Methanol-d_4) δ 177.0, 155.2, 151.6, 151.0, 145.0, 133.3, 129.8, 125.5, 72.3, 65.3, 60.8, 55.4, 45.5, 43.1, 34.1, 32.0, 31.7, 29.4, 29.4, 29.3, 29.3, 29.2, 29.1, 29.0, 29.0, 28.9, 27.7, 26.2, 25.5, 25.1, 24.6, 22.3, 22.3, 21.1, 13.0.

m/z (HRMS ESI): calcd for $C_{33}H_{56}N_5O_5^+$ $[M+H]^+$: 602.4276, found: 602.4269.

Synthesis of glucose analog 9

To a solution of **6-F** (10 mg, 0.04 mmol) in DMSO (1 mL), D-glucosamine hydrochloride (9 mg, 0.04 mmol) and triethylamine (17 µL, 0.12 mmol) were added, and the mixture was stirred at r.t. for 1.5 h. The mixture was poured into cold DCM and the precipitate was filtered and purified by semi-preparative HPLC.

(3R,4R,5S,6R)-6-(hydroxymethyl)-3-((7-nitrobenzo[c][1,2,5]selenadiazol-4-yl)amino)tetrahydro-2H-pyran-2,4,5-triol (90% yield, reddish solid) ^{1}H NMR (500 MHz, DMSO-d_6) δ 8.58 (d, J = 8.9 Hz, 1H), 7.06 (s, 1H), 6.61 (d, J = 8.9 Hz, 1H), 5.20 (d, J = 4.3 Hz, 2H), 5.09 (s, 1H), 4.52 (s, 1H), 3.77 (d, J = 10.5 Hz, 1H), 3.70 (dd, J = 10.3, 5.0 Hz, 1H), 3.61 – 3.53 (m, 1H). ^{13}C NMR (126 MHz, DMSO-d_6) δ 152.1, 148.5, 135.6, 128.7, 99.0, 90.6, 73.3, 73.0, 70.8, 61.5, 58.3.

m/z (HRMS ESI): calcd. for $C_{12}H_{15}N_4O_7Se^+$ $[M+H]^+$: 407.0092, found: 407.0100.

Synthesis of lactic acid analog 10

To a solution of **6-F** (20 mg, 0.08 mmol) in DMSO (0.5 mL), L-isoserine (17 mg, 0.16 mmol) was added, and the mixture was stirred at 50 ^{o}C for 4 h. The mixture was diluted with MeOH and purified by semi-preparative HPLC.

(*R*)-2-hydroxy-3-((7-nitrobenzo[c][1,2,5]selenadiazol-4-yl)amino)propanoic acid (38% yield, reddish solid) ^{1}H NMR (500 MHz, DMSO-d_6) δ 8.60 (d, J = 8.9, 1H), 7.99 (t, J = 6.0, 1H), 6.48 (d, J = 9.0, 1H), 4.38 (dd, J = 7.2, 4.5, 1H), 3.82 – 3.59 (m, 2H). ^{13}C NMR (126 MHz, DMSO-d_6) δ 174.2, 152.3, 148.7, 135.5, 129.0, 98.4, 69.1, 46.8, 40.9.

m/z (HRMS ESI): calcd. for $C_9H_9N_4O_5Se^+$ $[M+H]^+$: 332.9727, found: 332.9769

Synthesis of glucose analog 11

To a solution of **5-F** (27 mg, 0.14 mmol) in MeCN (0.8 mL) was added D-glucosamine hydrochloride (35 mg, 0.16 mmol) in saturated $NaHCO_{3\ (aq)}$ (0.8 mL) and the reaction was stirred at 30 °C for 24 h. The solvent was removed under reduced pressure, and the product was isolated by normal phase chromatography (DCM:MeOH, 9:1).

(3R,4R,5S,6R)-6-(hydroxymethyl)-3-((7-nitrobenzo[c][1,2,5]thiadiazol-4-yl)amino)tetrahydro-2H-pyran-2,4,5-triol (13% yield, orange solid) ^{1}H NMR (500 MHz, DMSO-d_6) δ 8.61 (d, J = 7.4 Hz, 1H), 7.03 (s, 1H), 6.82 (d, J = 7.4 Hz, 1H), 5.21 (s, 1H), 5.07 – 5.18 (m, 2H), 4.52 (t, 1H), 3.85 – 3.65 (m, 4H), 3.50 – 3.47 (m, 1H). ^{13}C NMR (126 MHz, DMSO-d_6) δ 149.2, 146.6, 134.6, 125.7, 100.9, 95.81, 90.3, 77.1, 74.9, 72.6, 71.0, 61.4, 58.2.

m/z (HRMS ESI): calcd. for $C_{12}H_{15}N_4O_7S^+$ $[M+H]^+$: 359.0656, found: 359.0687.

Synthesis of lipid analog Cer-Fluor

A solution of HOBt (4.8 mg, 36 µmol), 6-[fluorescein-5(6)-carboxamido]hexanoic acid (15 mg, 30 µmol) and EDC (7.7 mg, 40 µmol) in anhydrous DMF (0.5 mL) was added dropwise to a solution of sphingosine (9 mg, 30 µmol) and triethylamine (15 µL, 107 µmol) under Ar atmosphere. The reaction mixture was stirred at r.t. for 3 h and concentrated *in vacuo*. The resulting crude was purified by flash chromatography in XAD4 resin and eluted with H_2O-acetonitrile (from 0% to 100% acetonitrile). Fractions eluted in 20-80% acetonitrile were combined, evaporated to dryness, and further purified by normal chromatography in DCM-MeOH (8:2) to isolate the compound as an orange solid (1.6 mg, 7% yield).

m/z (HRMS ESI): calcd. for $C_{45}H_{59}N_2O_9^+$ $[M+H]^+$: 771.4215, found: 771.4235.

2. NMR Spectra

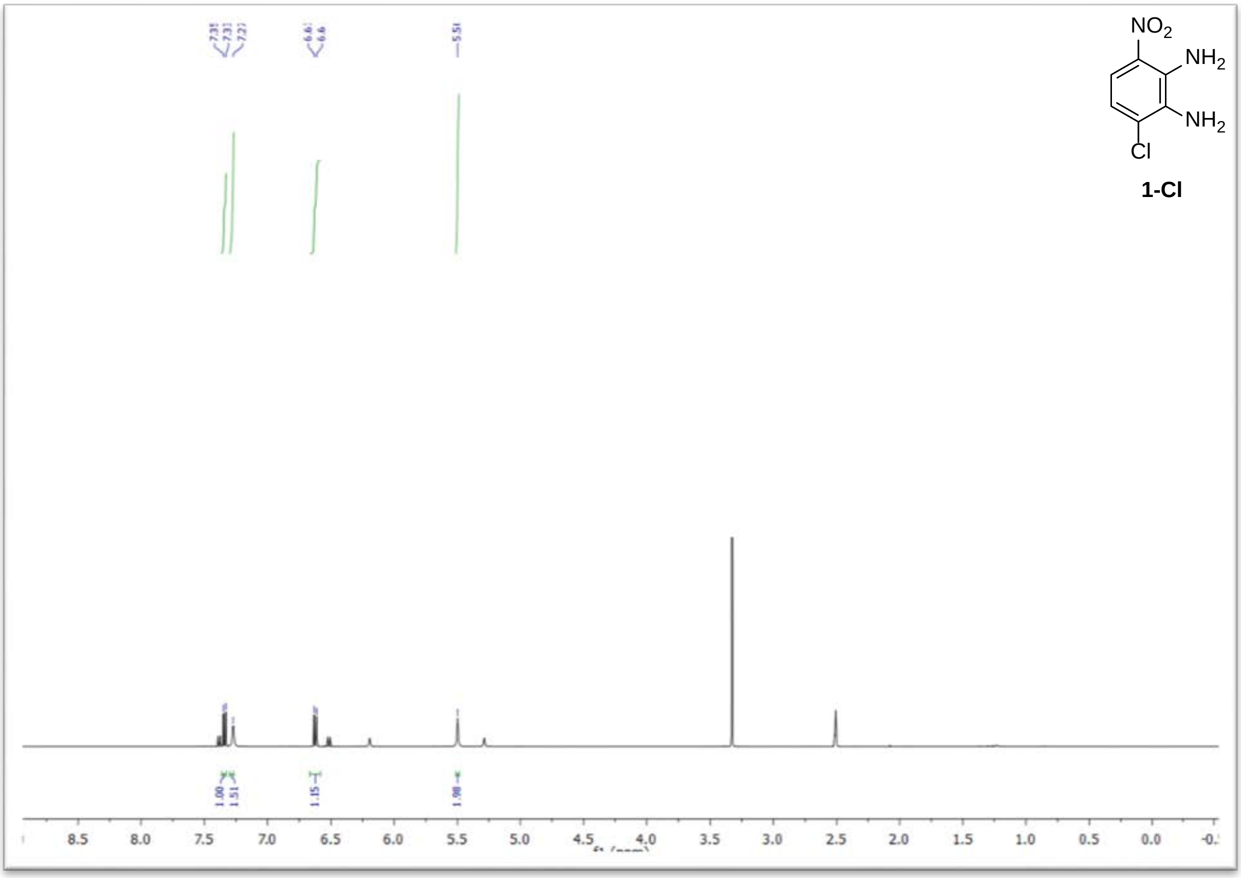

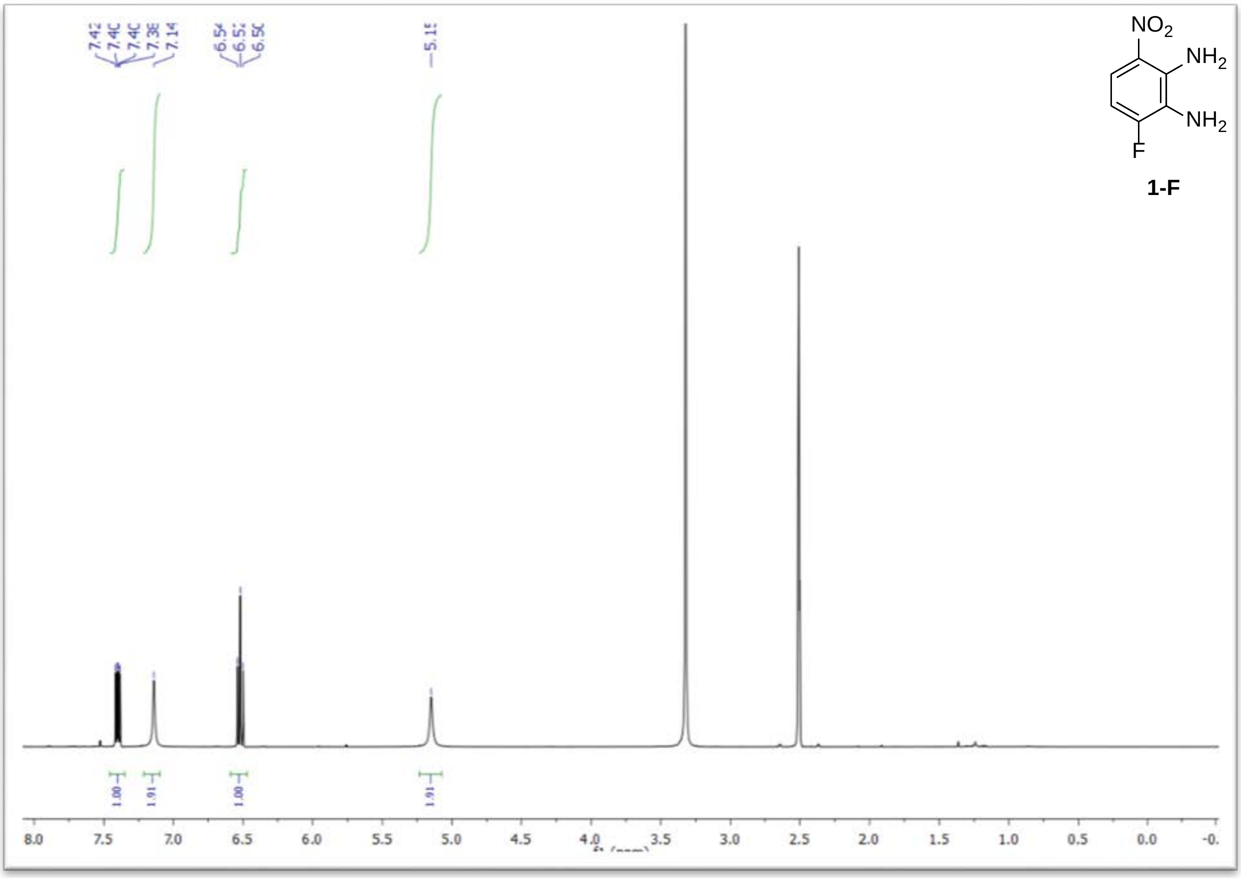

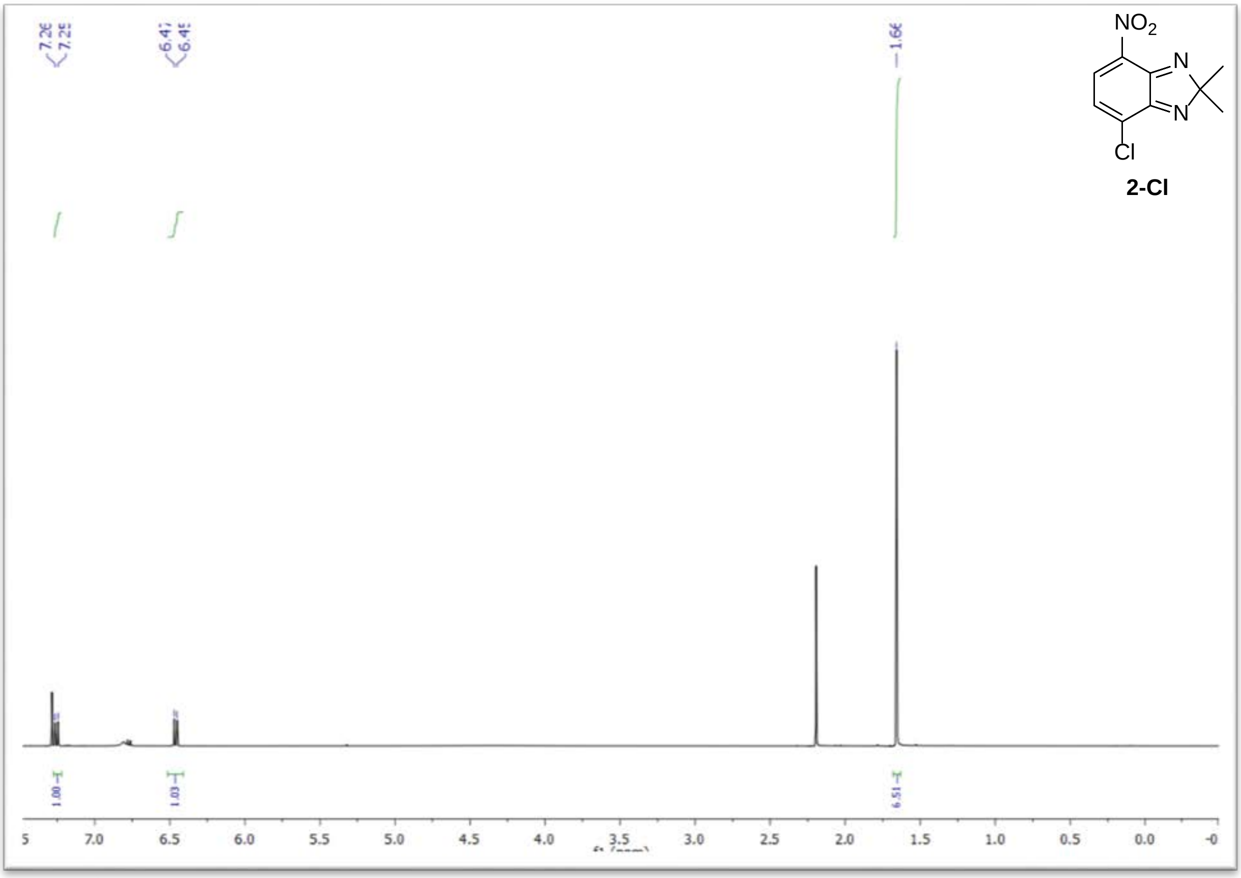

NO2
N
N
Cl
2-Cl
7.26
7.25
6.47
6.45
1.66
1.00
1.03
6.51
7.0
6.5
6.0
5.5
5.0
4.5
4.0
3.5
3.0
2.5
2.0
1.5
1.0
0.5
0.0

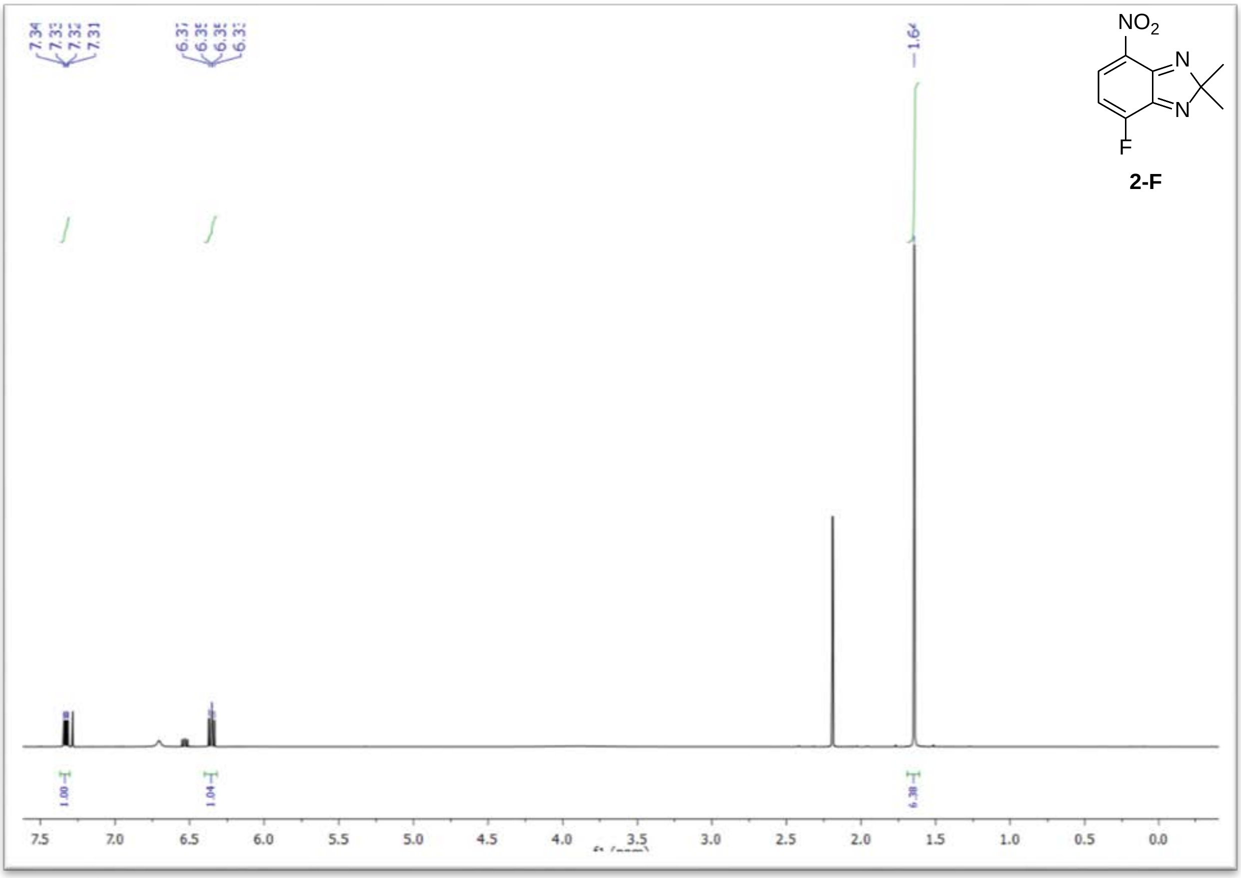

NO2
N
N
F
2-F
7.34
1.64
1.00
1.04
6.38
7.5
7.0
6.5
6.0
5.5
5.0
4.5
4.0
3.5
3.0
2.5
2.0
1.5
1.0
0.5
0.0

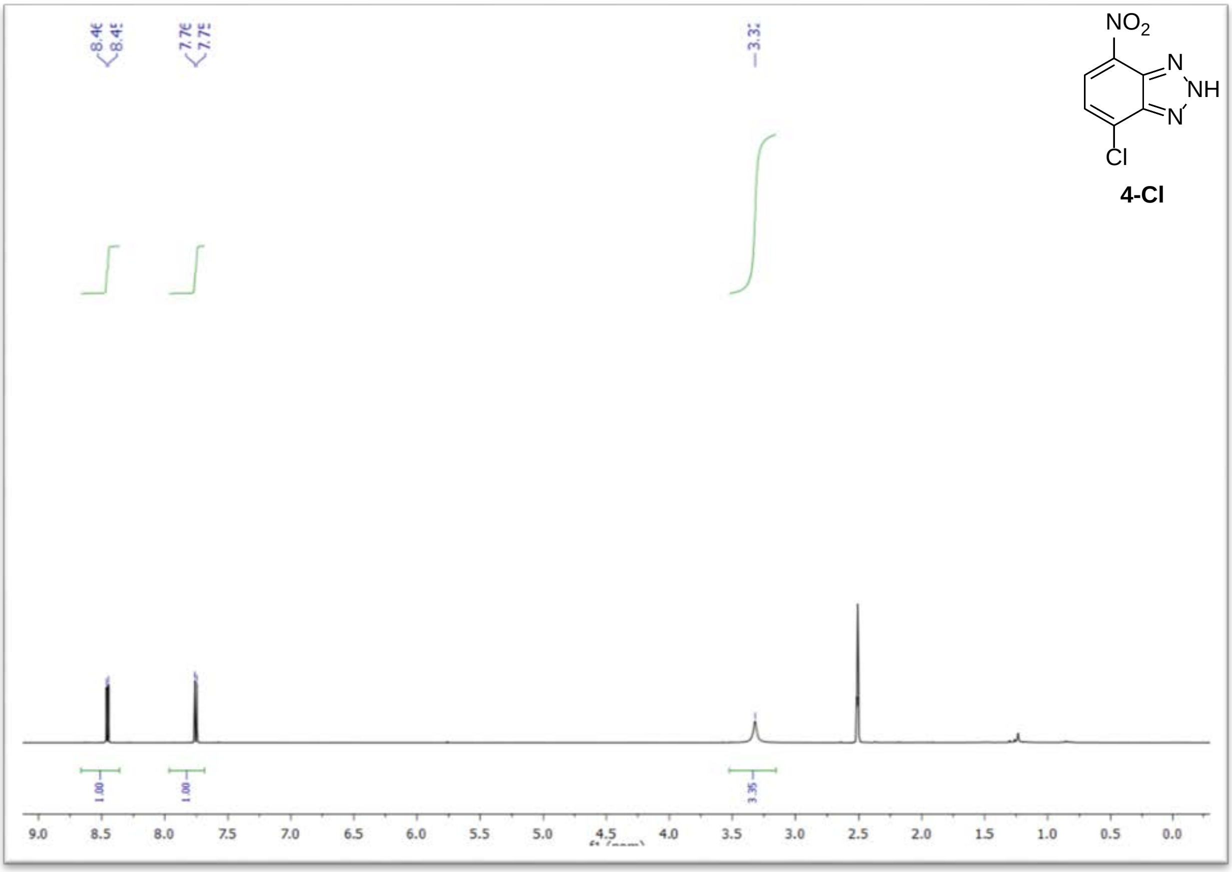

4-Cl

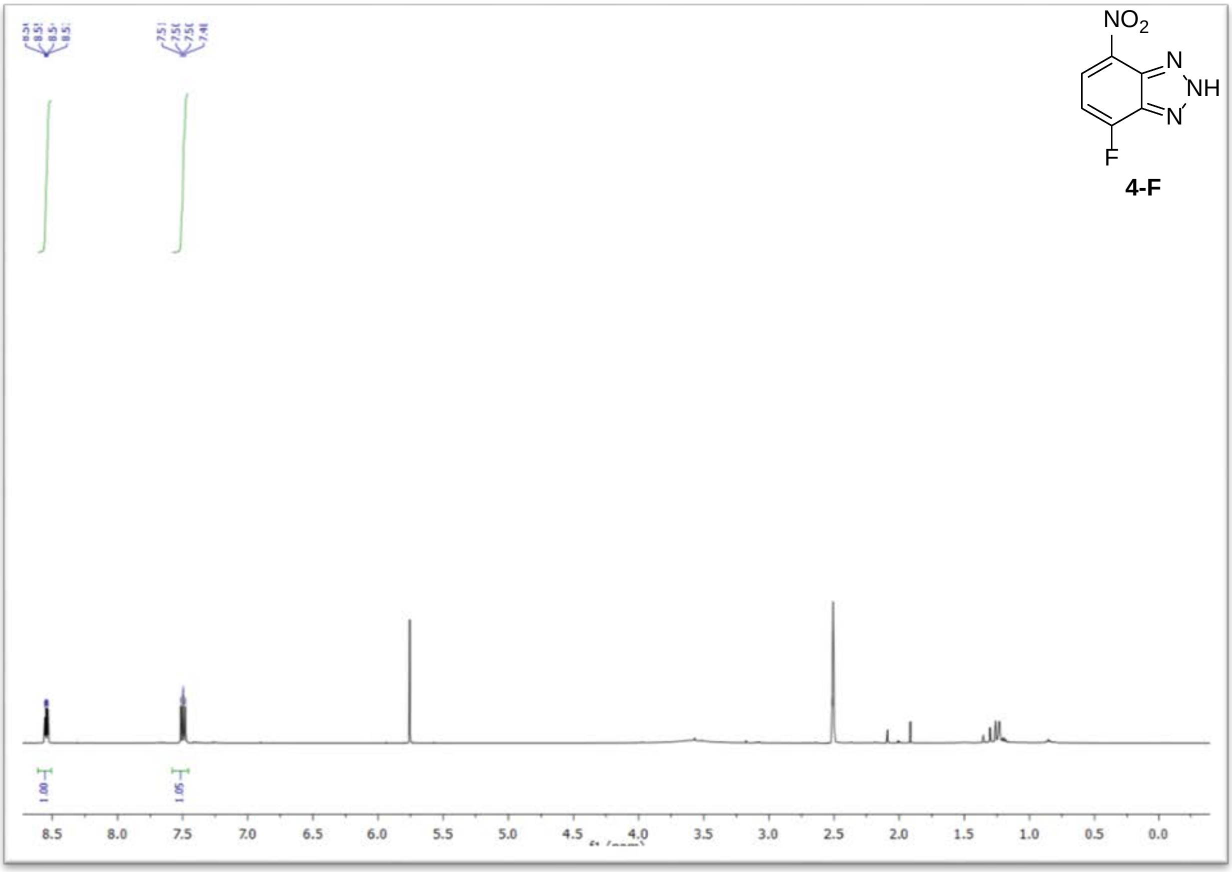

4-F

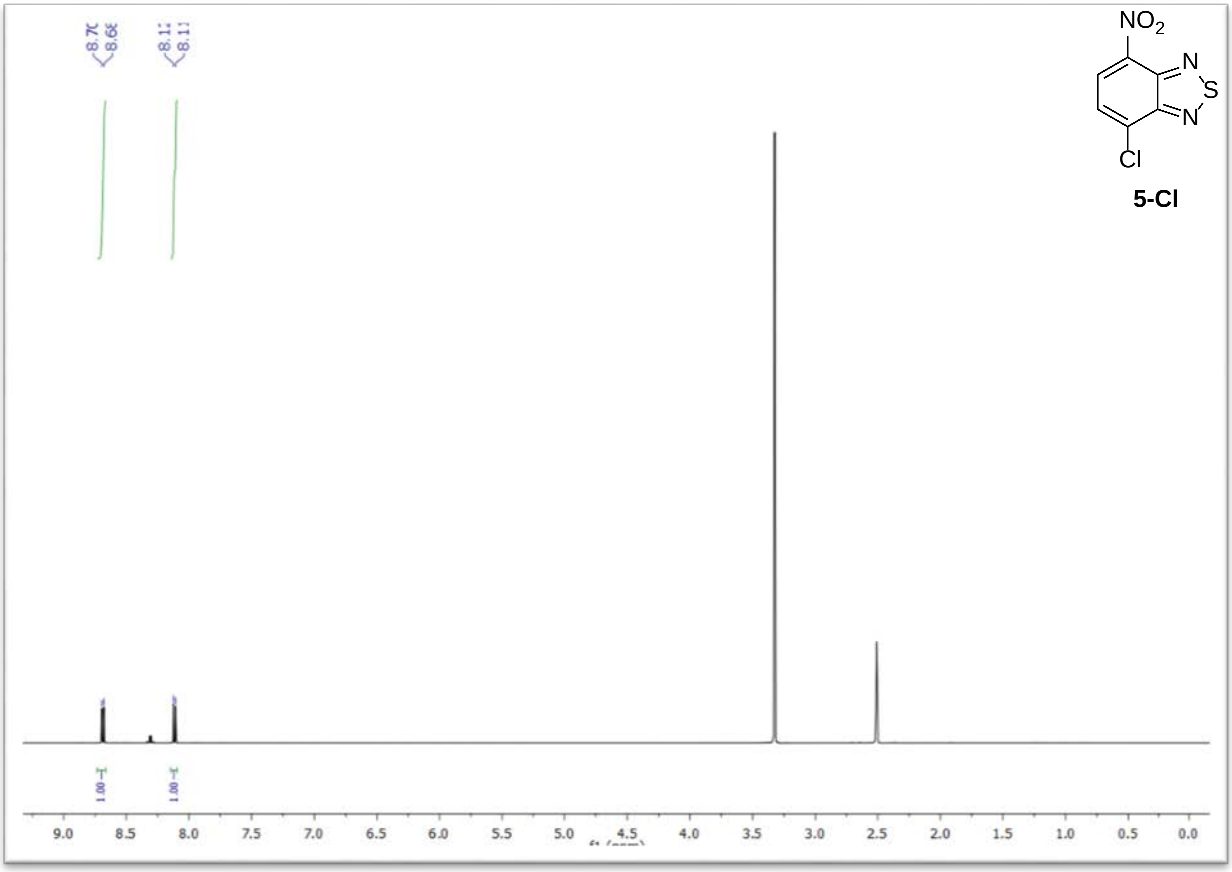
NO2
N
S
N
Cl
8.70
8.69
8.12
8.11
1.00
1.00
9.0 8.5 8.0 7.5 7.0 6.5 6.0 5.5 5.0 4.5 4.0 3.5 3.0 2.5 2.0 1.5 1.0 0.5 0.0

5-Cl

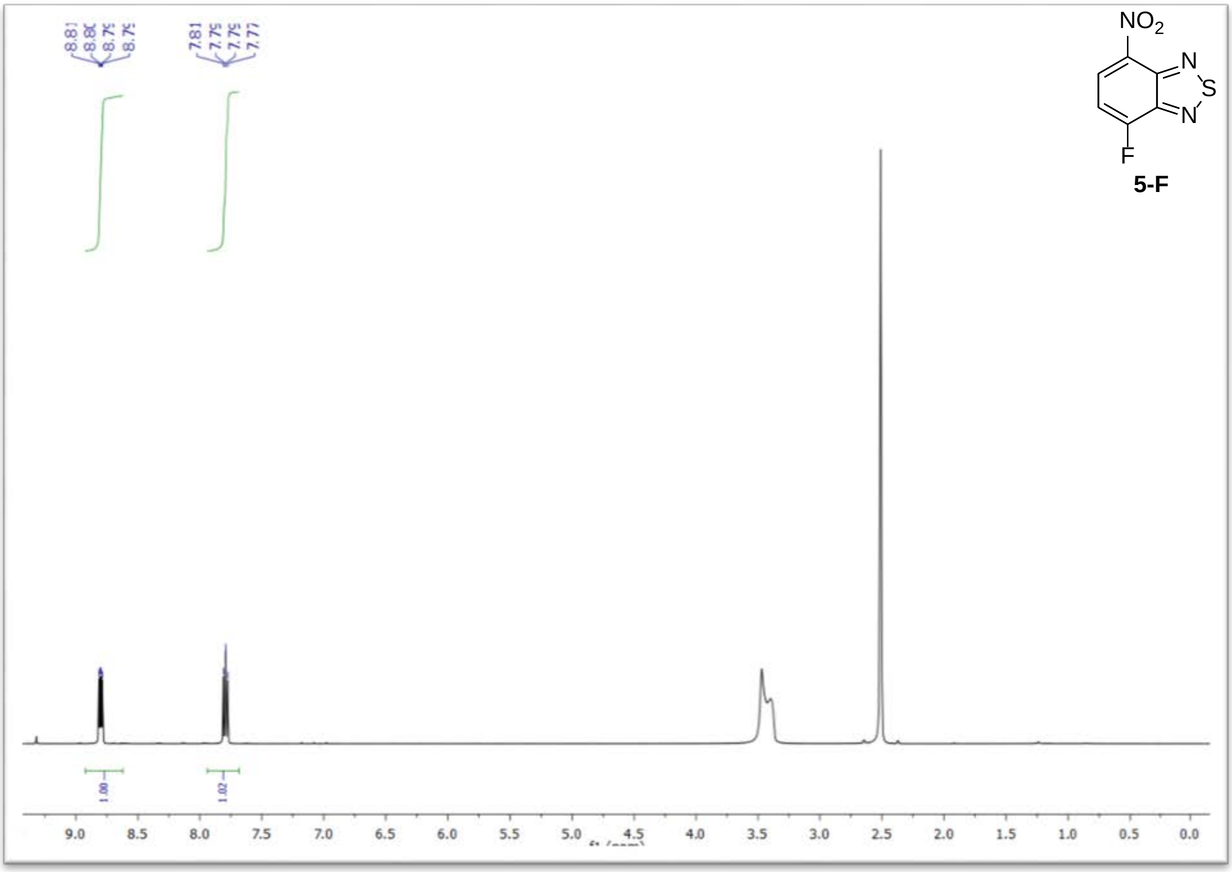
NO2
N
S
N
F
7.81
7.79
7.79
7.77
1.00
1.02
9.0 8.5 8.0 7.5 7.0 6.5 6.0 5.5 5.0 4.5 4.0 3.5 3.0 2.5 2.0 1.5 1.0 0.5 0.0

5-F

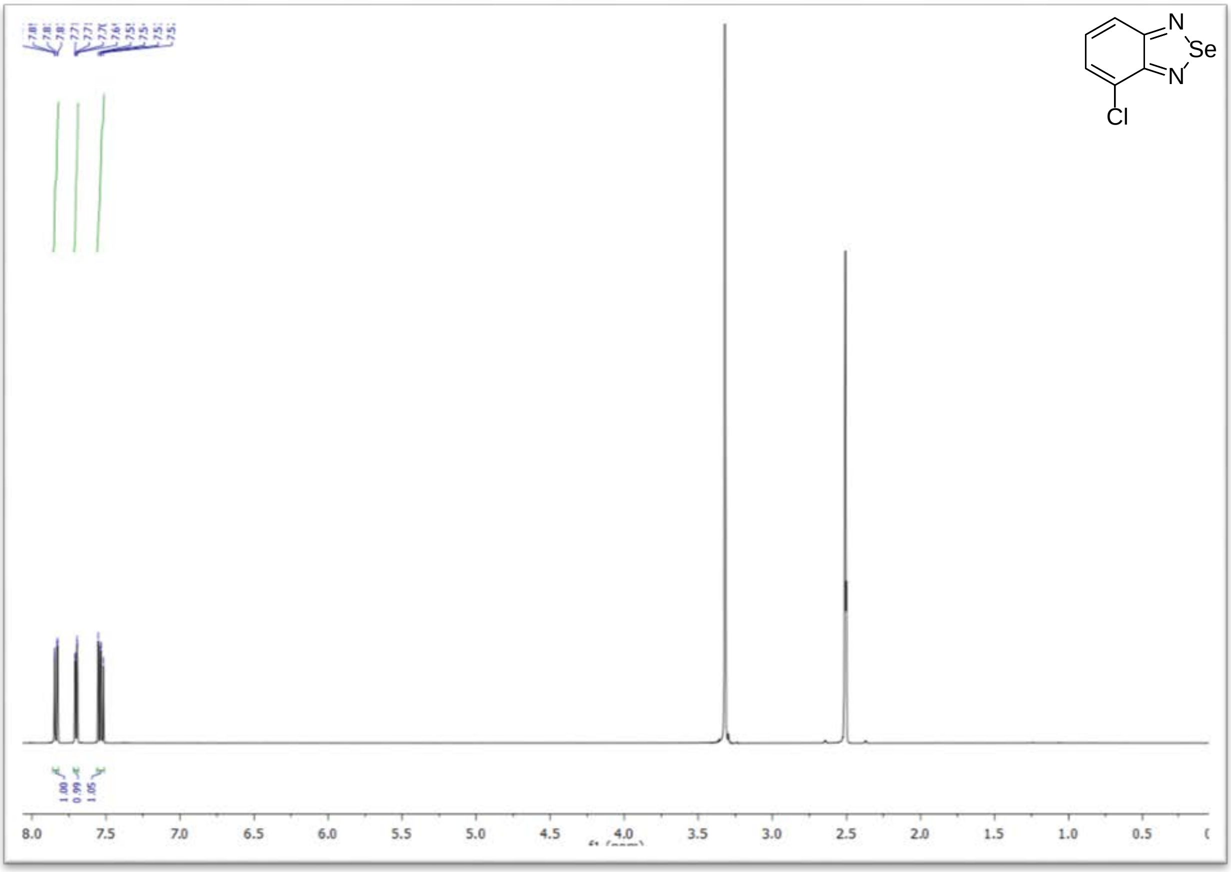

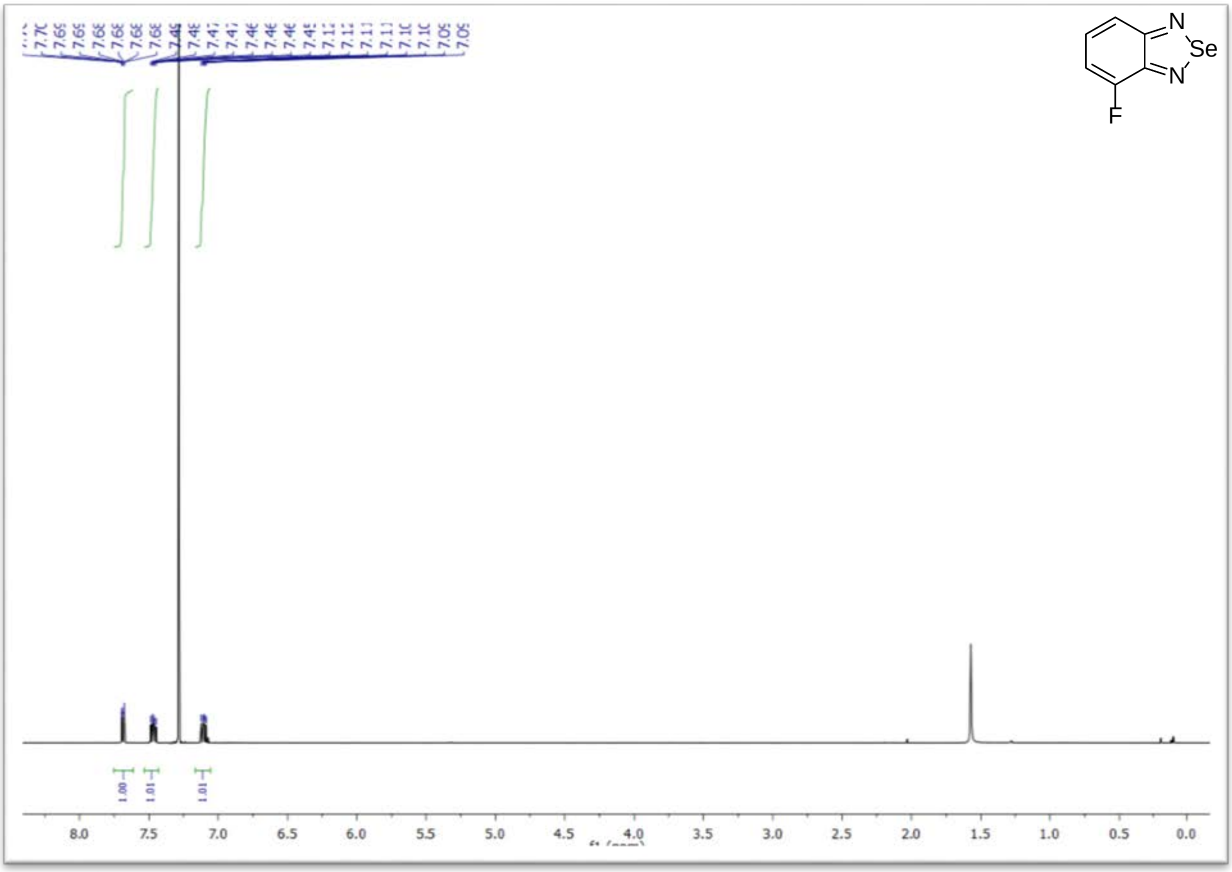

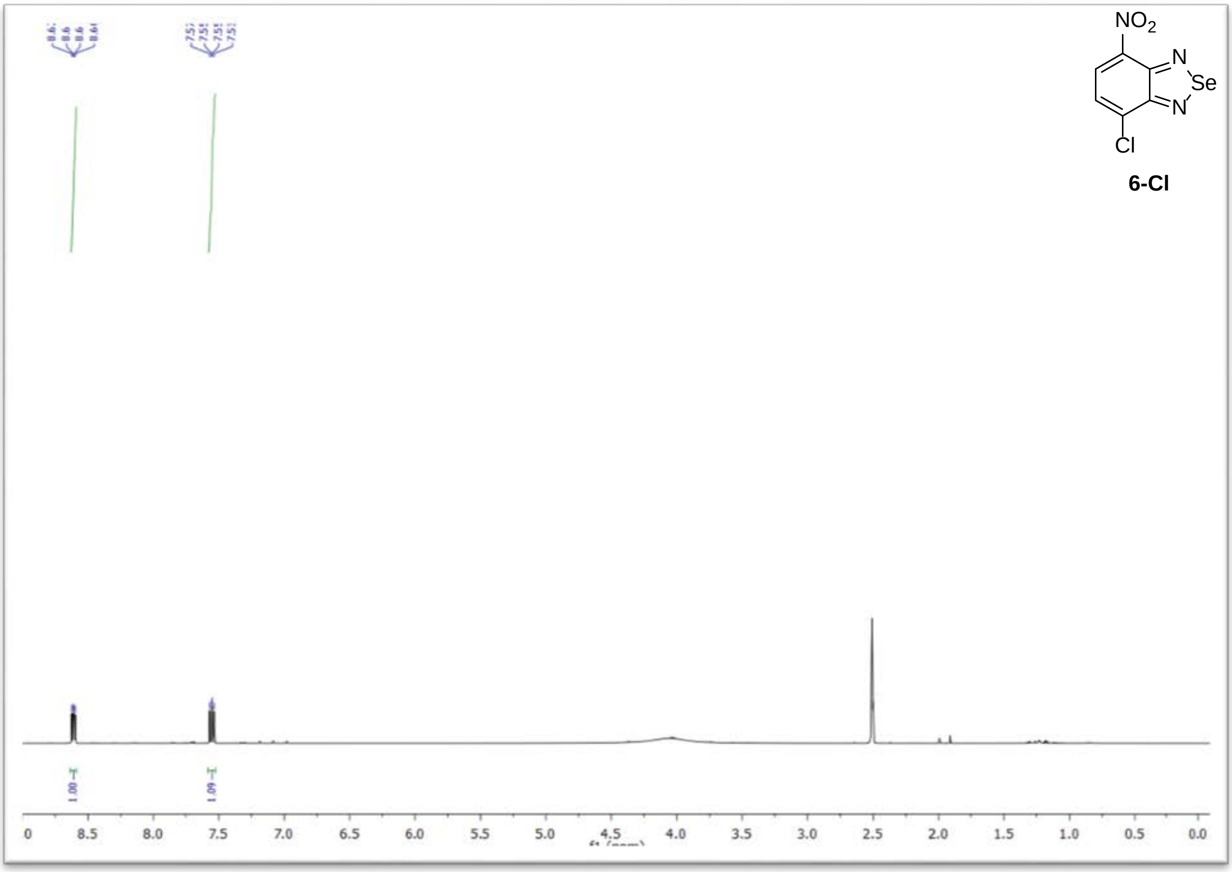
NO2
N
Se
N
Cl
8.5
8.0
7.5
7.0
6.5
6.0
5.5
5.0
4.5
4.0
3.5
3.0
2.5
2.0
1.5
1.0
0.5
0.0

6-Cl

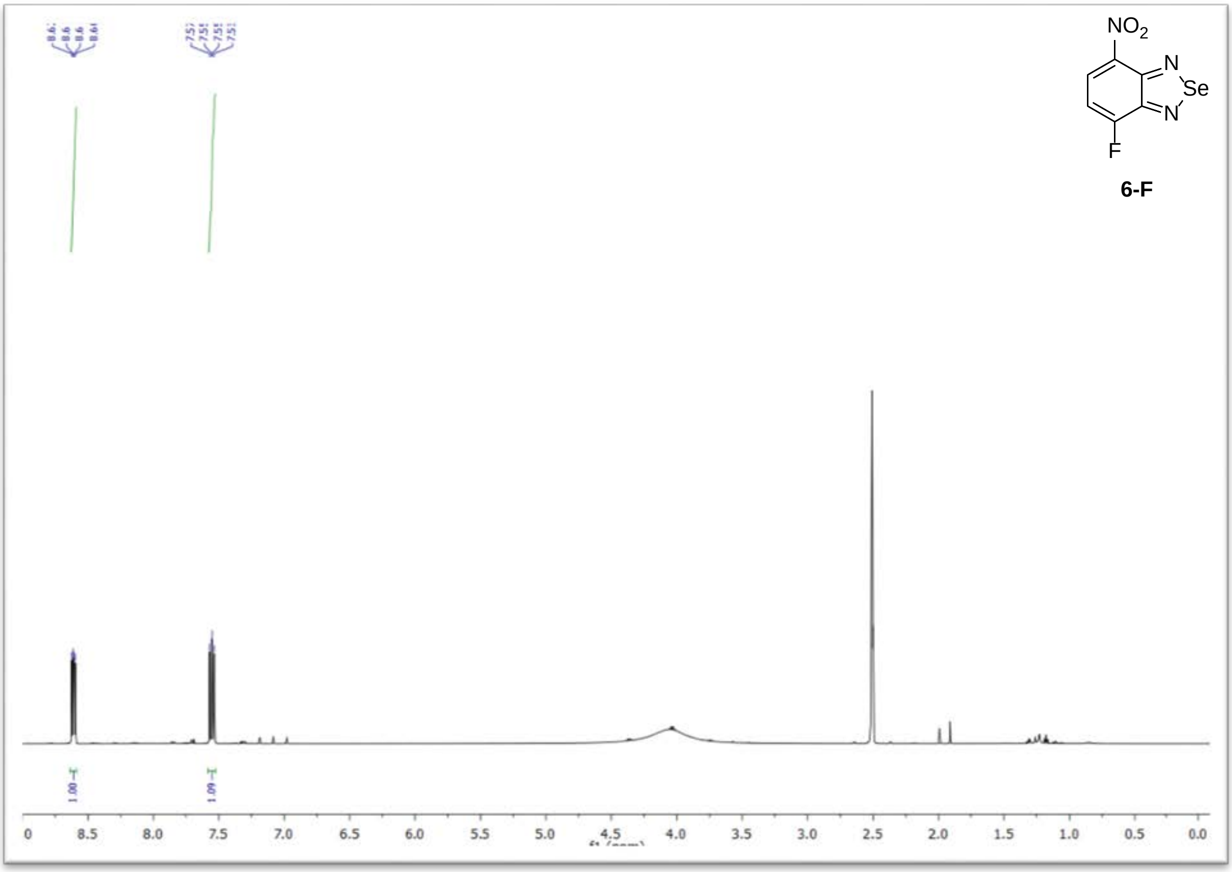
NO2
N
Se
N
F
8.5
8.0
7.5
7.0
6.5
6.0
5.5
5.0
4.5
4.0
3.5
3.0
2.5
2.0
1.5
1.0
0.5
0.0

6-F

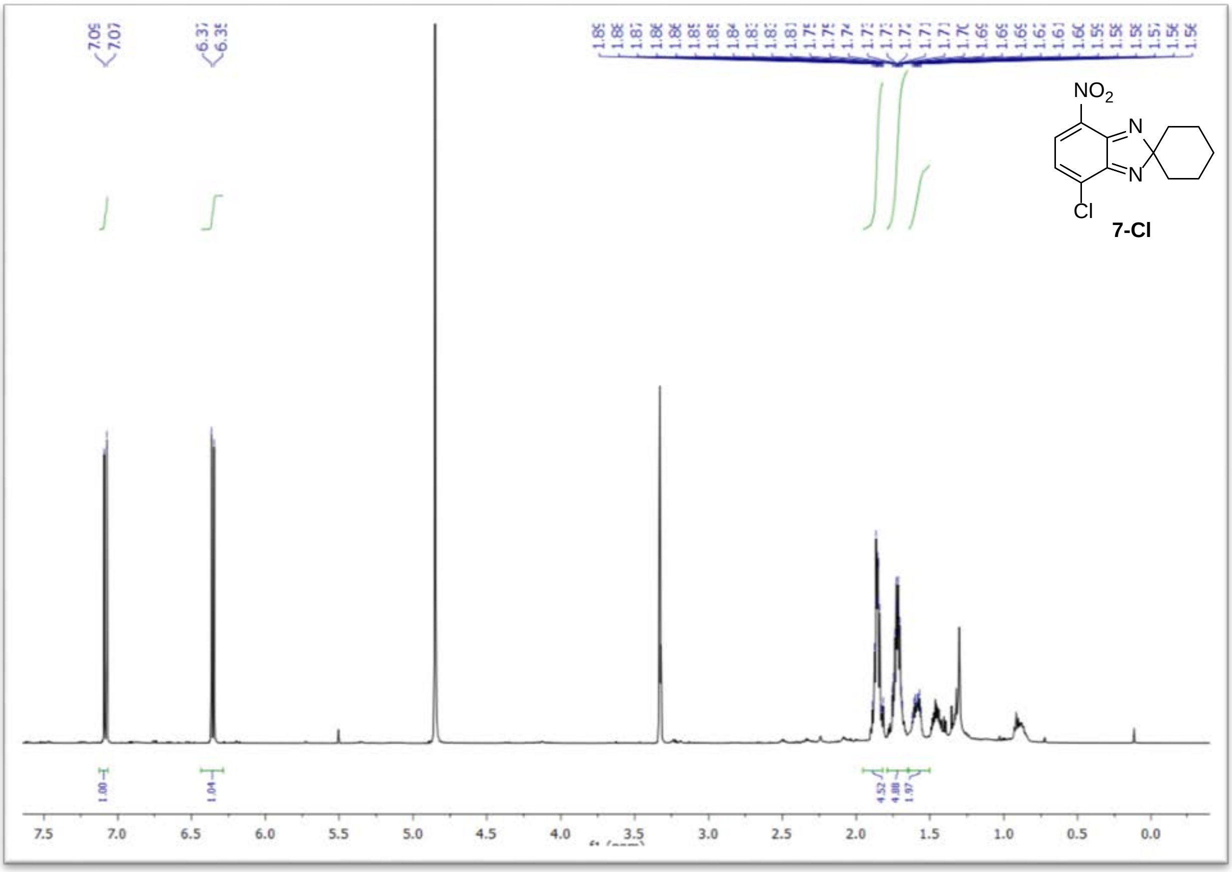
NO2
N
N
Cl
7-Cl
7.5
7.0
6.5
6.0
5.5
5.0
4.5
4.0
3.5
3.0
2.5
2.0
1.5
1.0
0.5
0.0

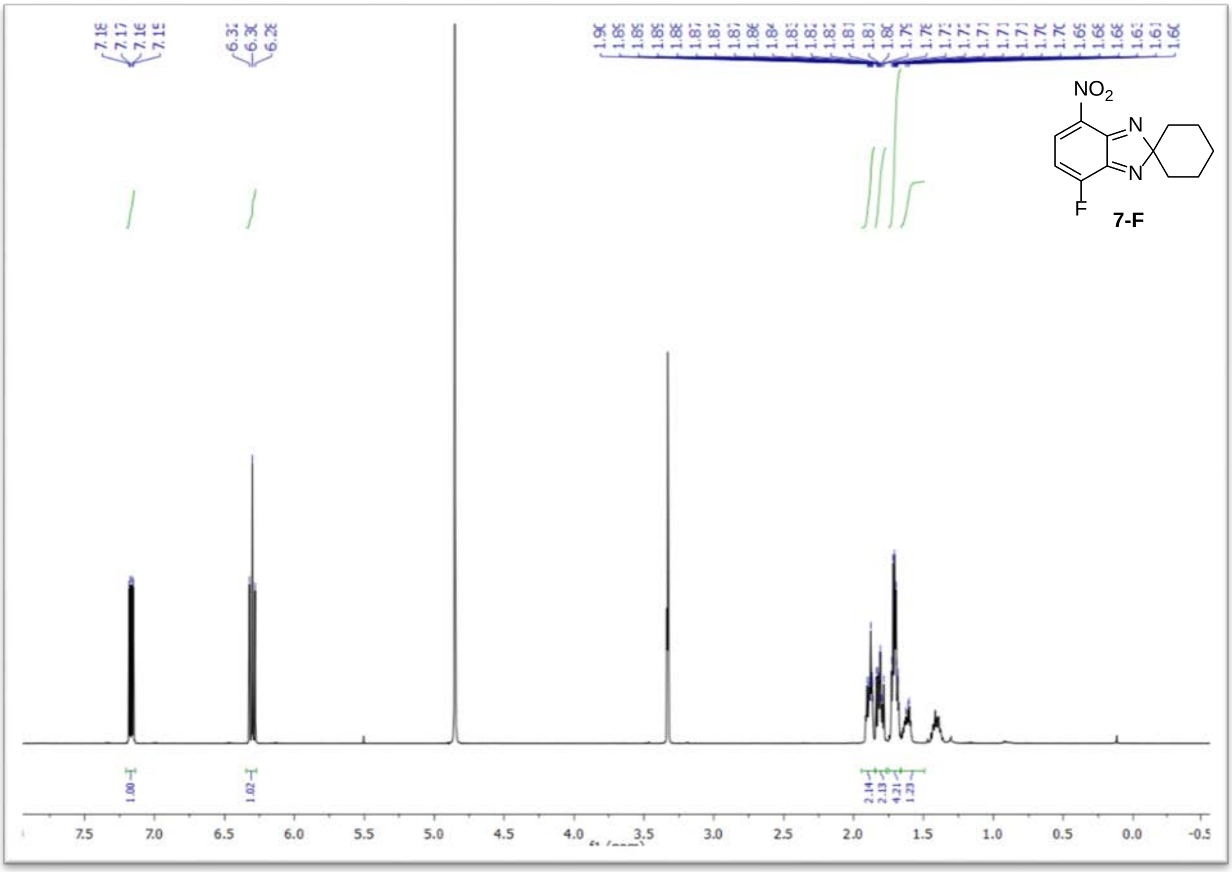
NO2
N
N
F
7-F
7.5
7.0
6.5
6.0
5.5
5.0
4.5
4.0
3.5
3.0
2.5
2.0
1.5
1.0
0.5
0.0
-0.5

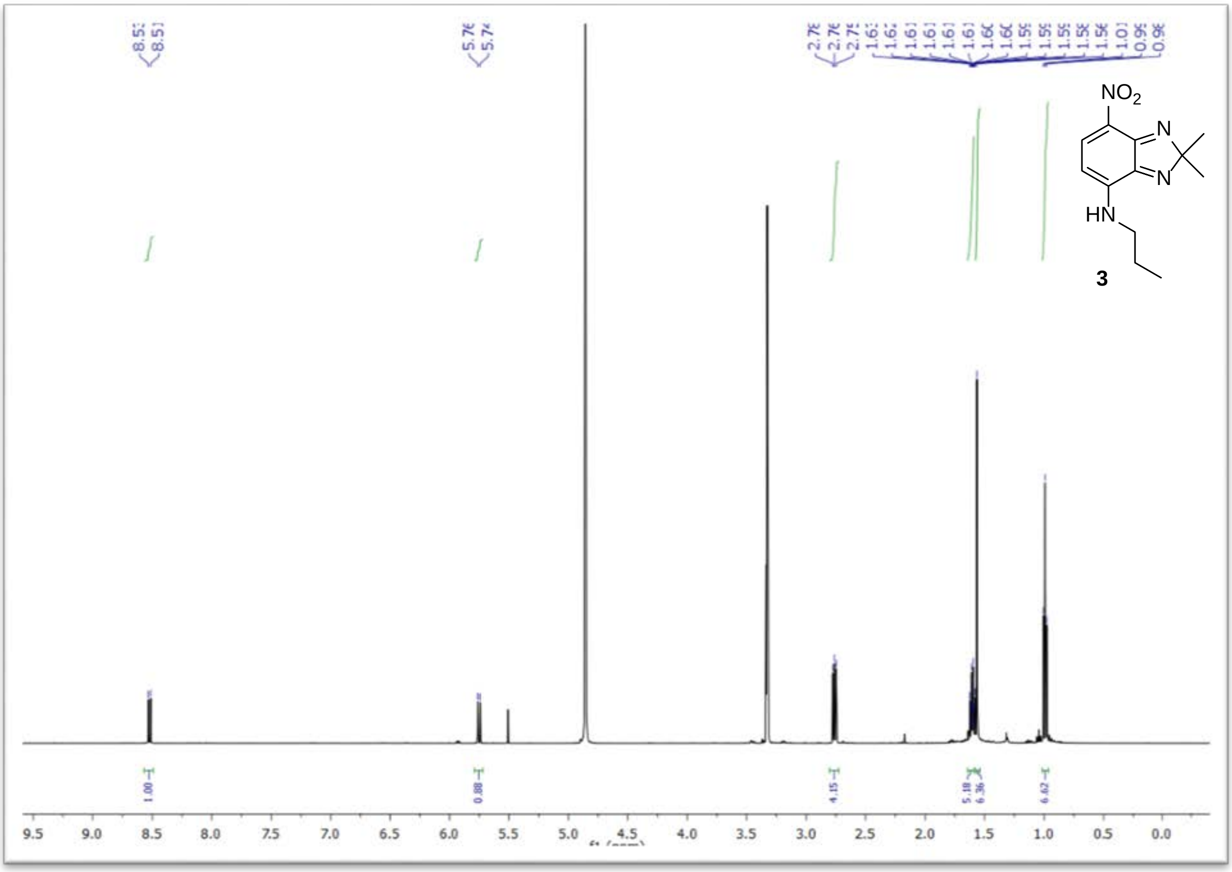

NO2
N
N
HN
3
1.00
0.88
4.15
5.18
6.36
6.62
9.5
9.0
8.5
8.0
7.5
7.0
6.5
6.0
5.5
5.0
4.5
4.0
3.5
3.0
2.5
2.0
1.5
1.0
0.5
0.0

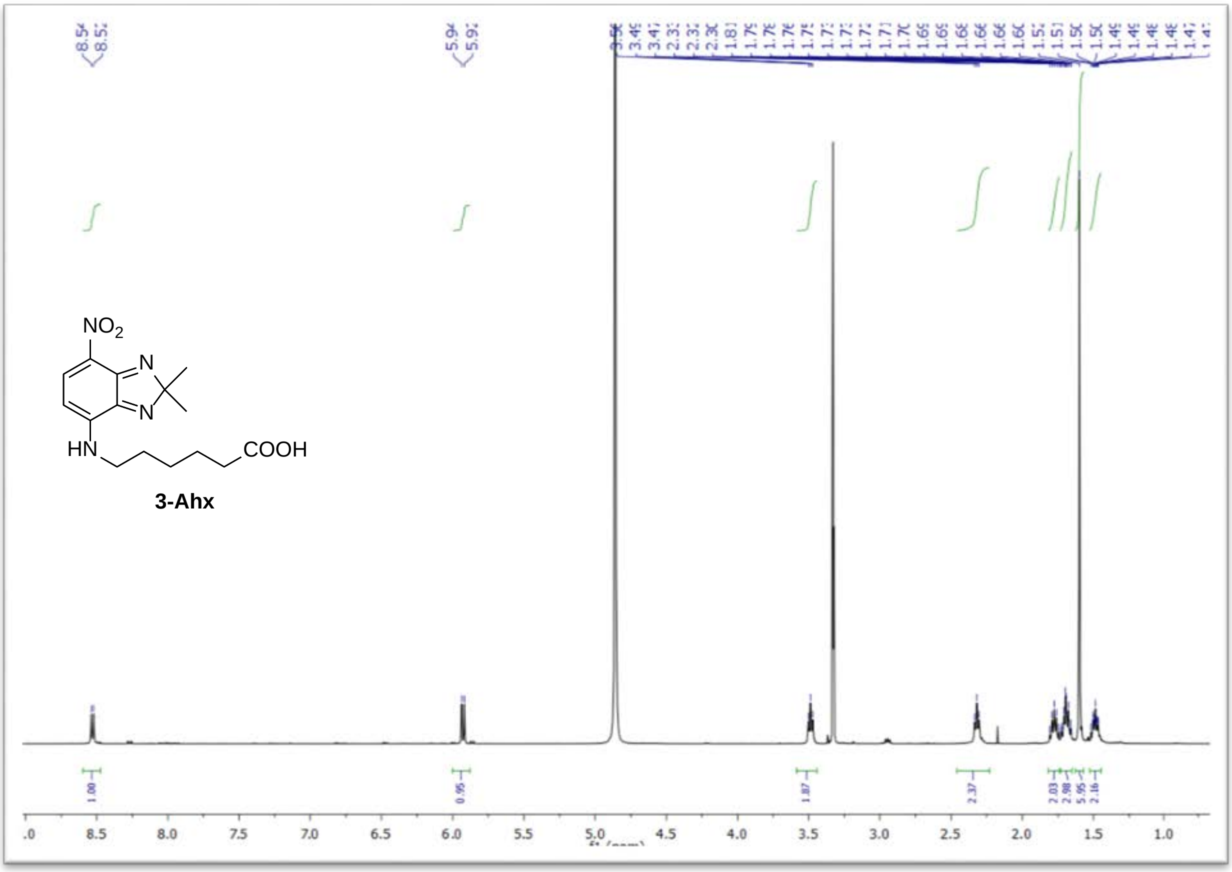

NO2
N
N
HN
COOH
3-Ahx
1.00
0.95
1.87
2.37
8.5
8.0
7.5
7.0
6.5
6.0
5.5
5.0
4.5
4.0
3.5
3.0
2.5
2.0
1.5
1.0

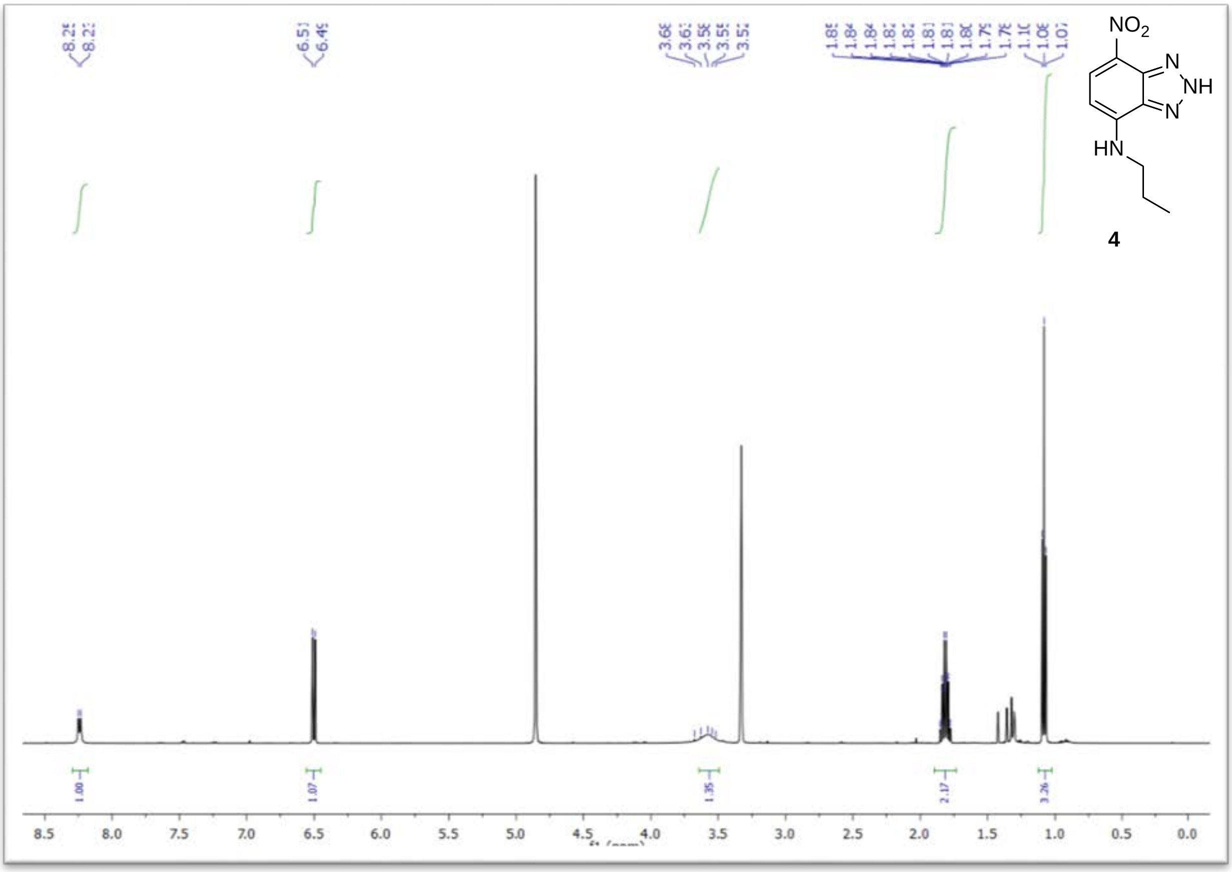

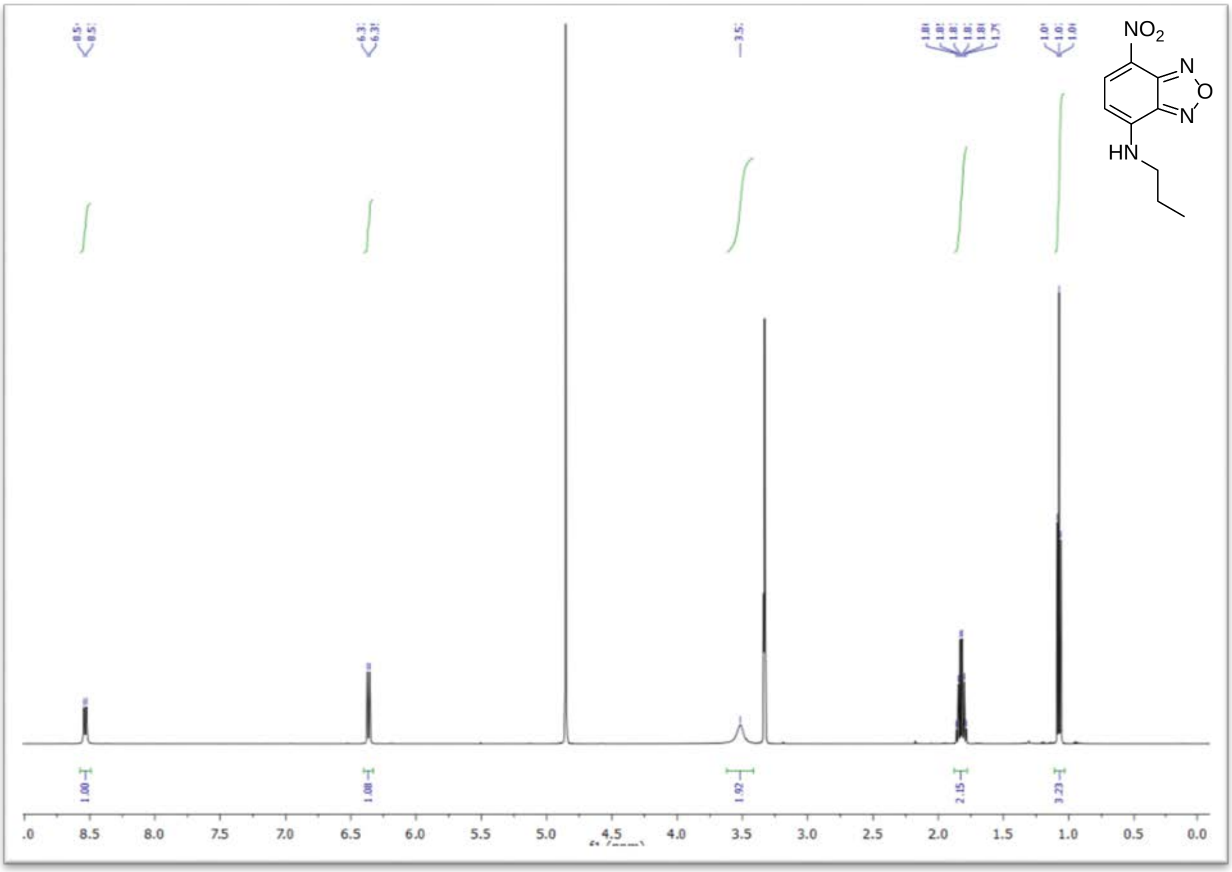

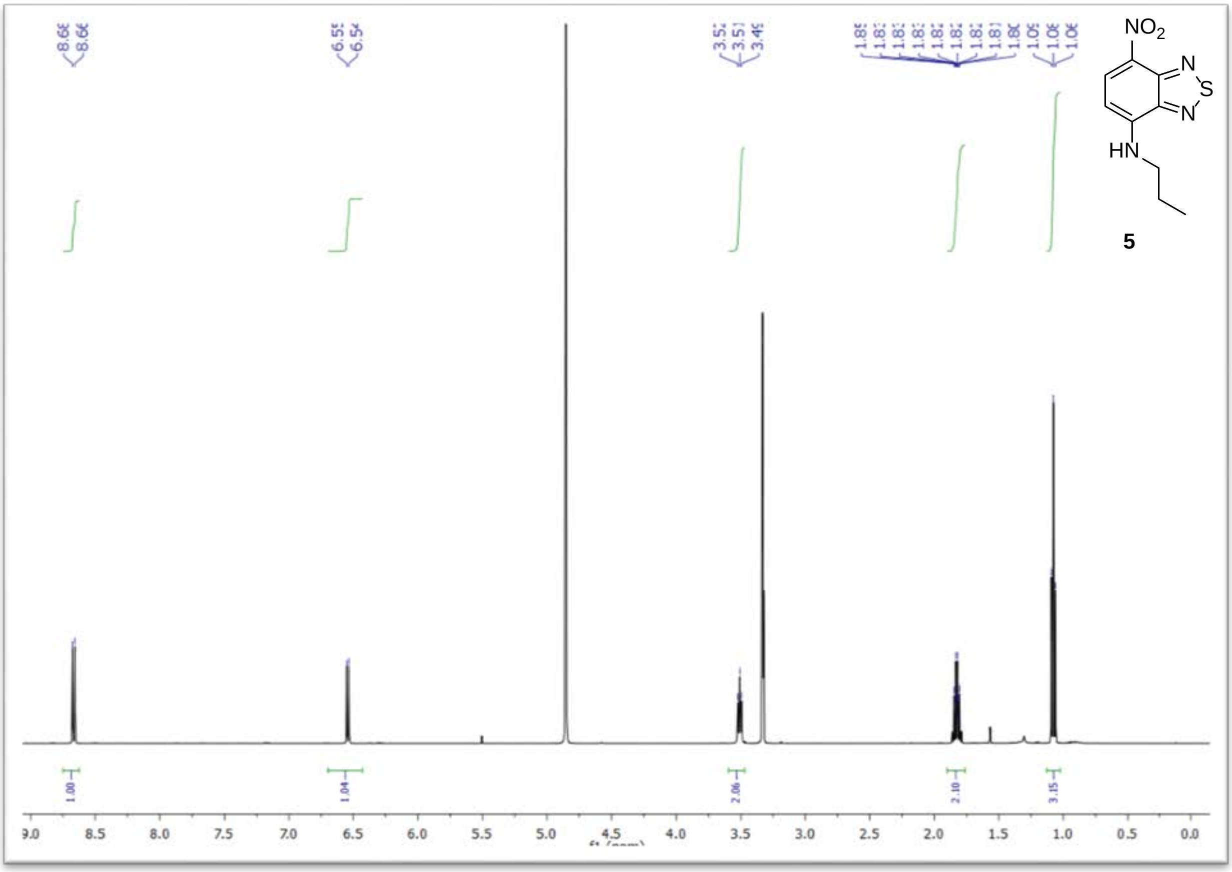
NO2
N
S
N
HN
5

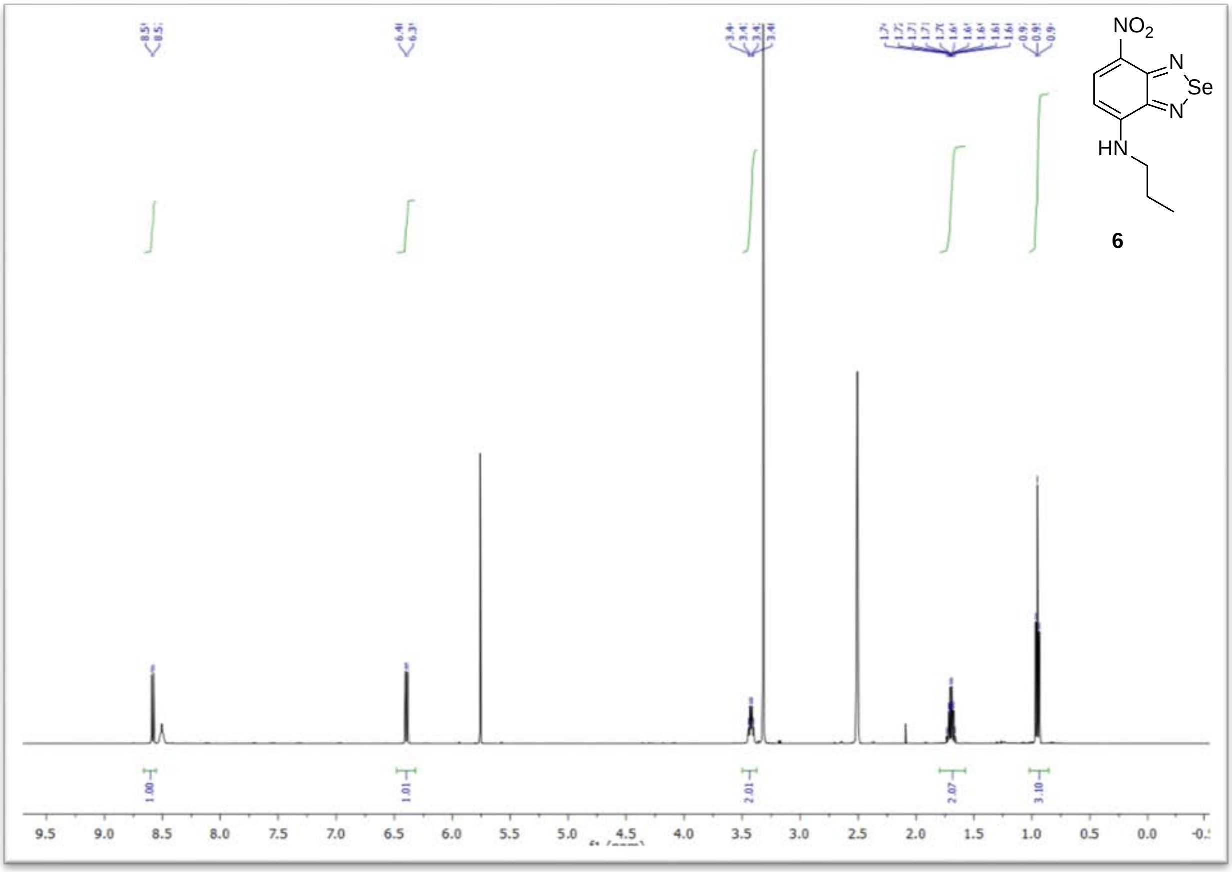
NO2
N
Se
N
HN
6

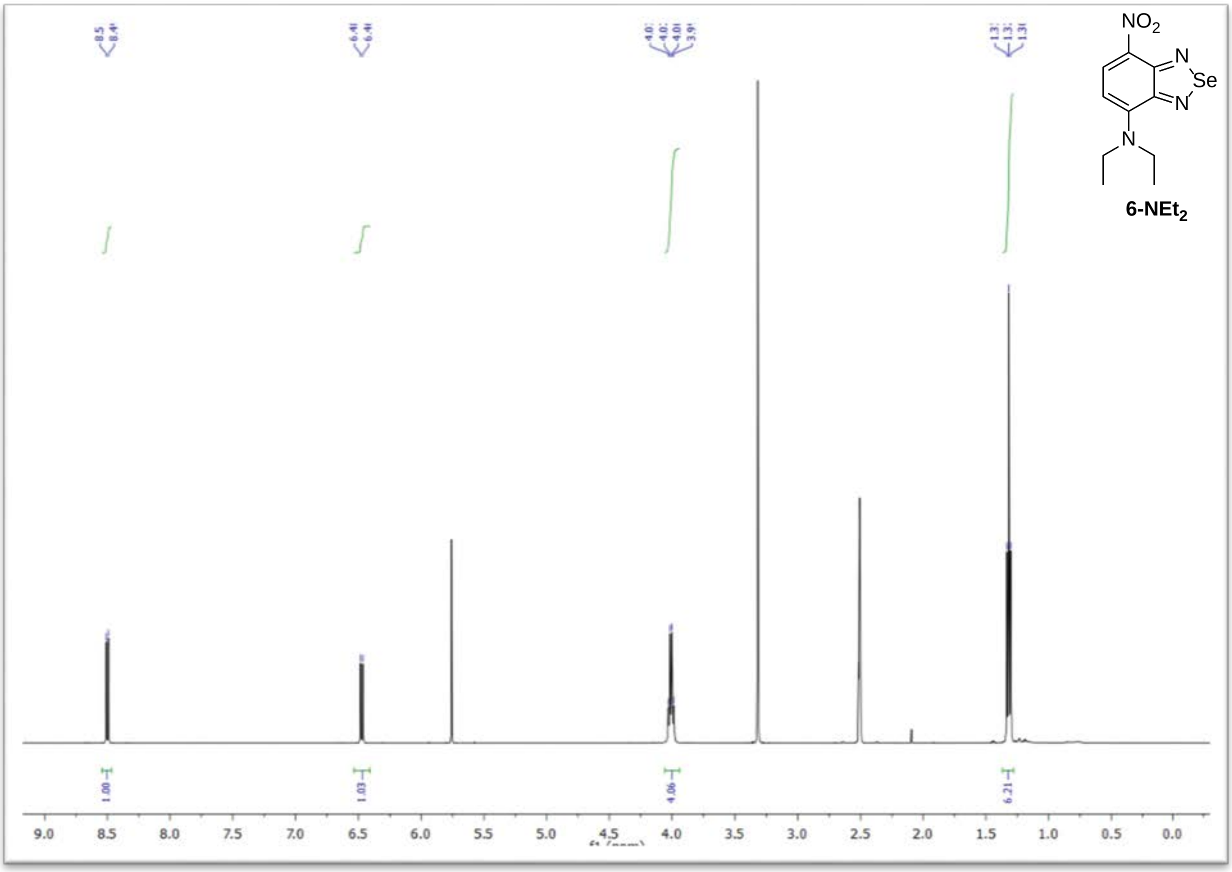

NO2
N
Se
N
N
6-NEt2
9.0 8.5 8.0 7.5 7.0 6.5 6.0 5.5 5.0 4.5 4.0 3.5 3.0 2.5 2.0 1.5 1.0 0.5 0.0

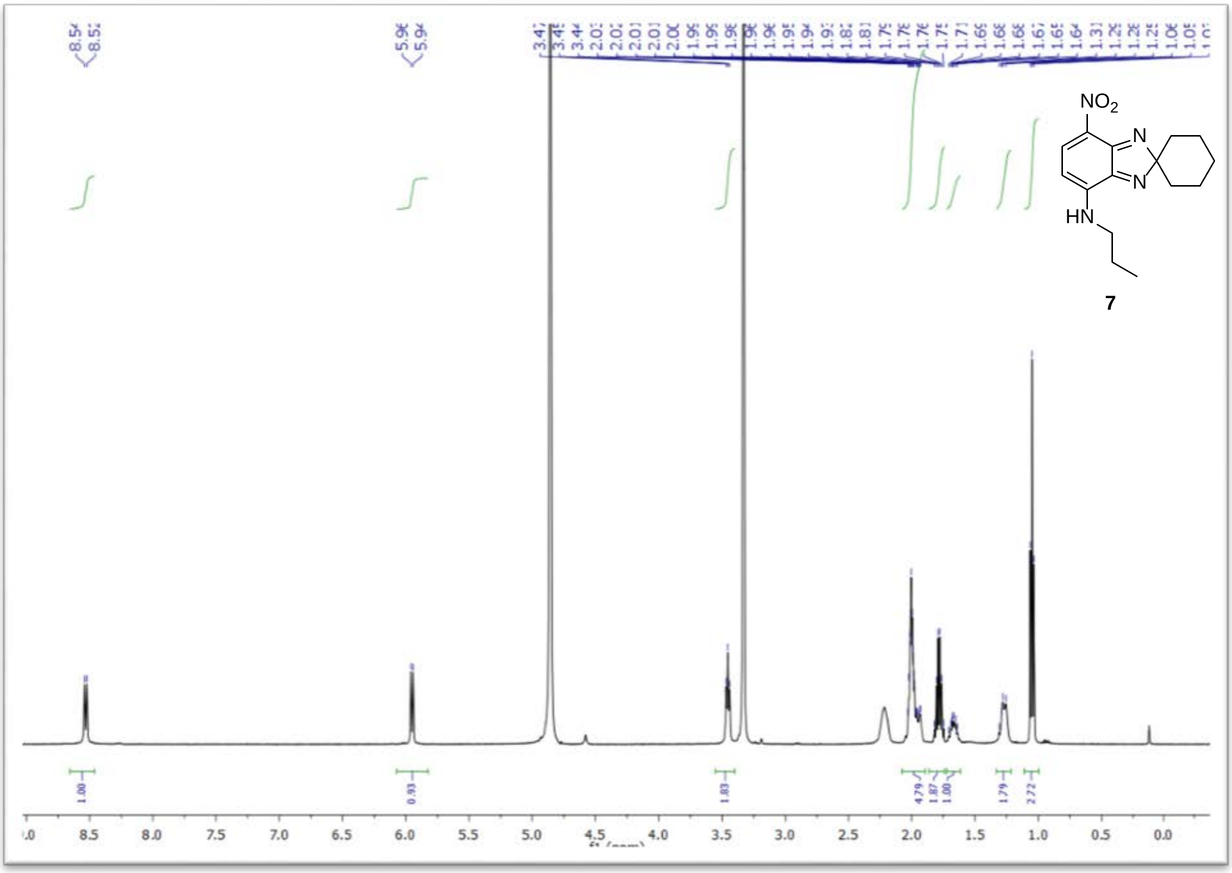

NO2
N
N
HN
7
8.5 8.0 7.5 7.0 6.5 6.0 5.5 5.0 4.5 4.0 3.5 3.0 2.5 2.0 1.5 1.0 0.5 0.0

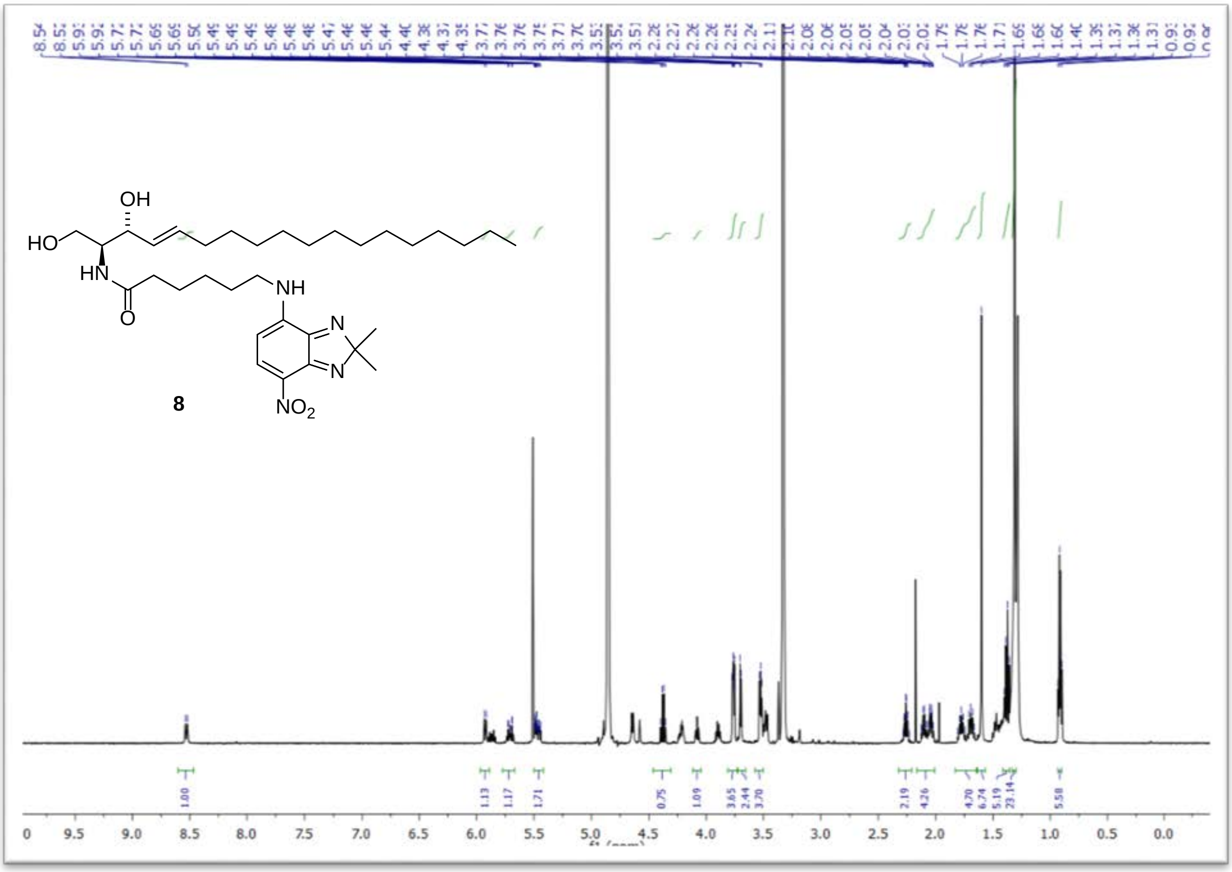

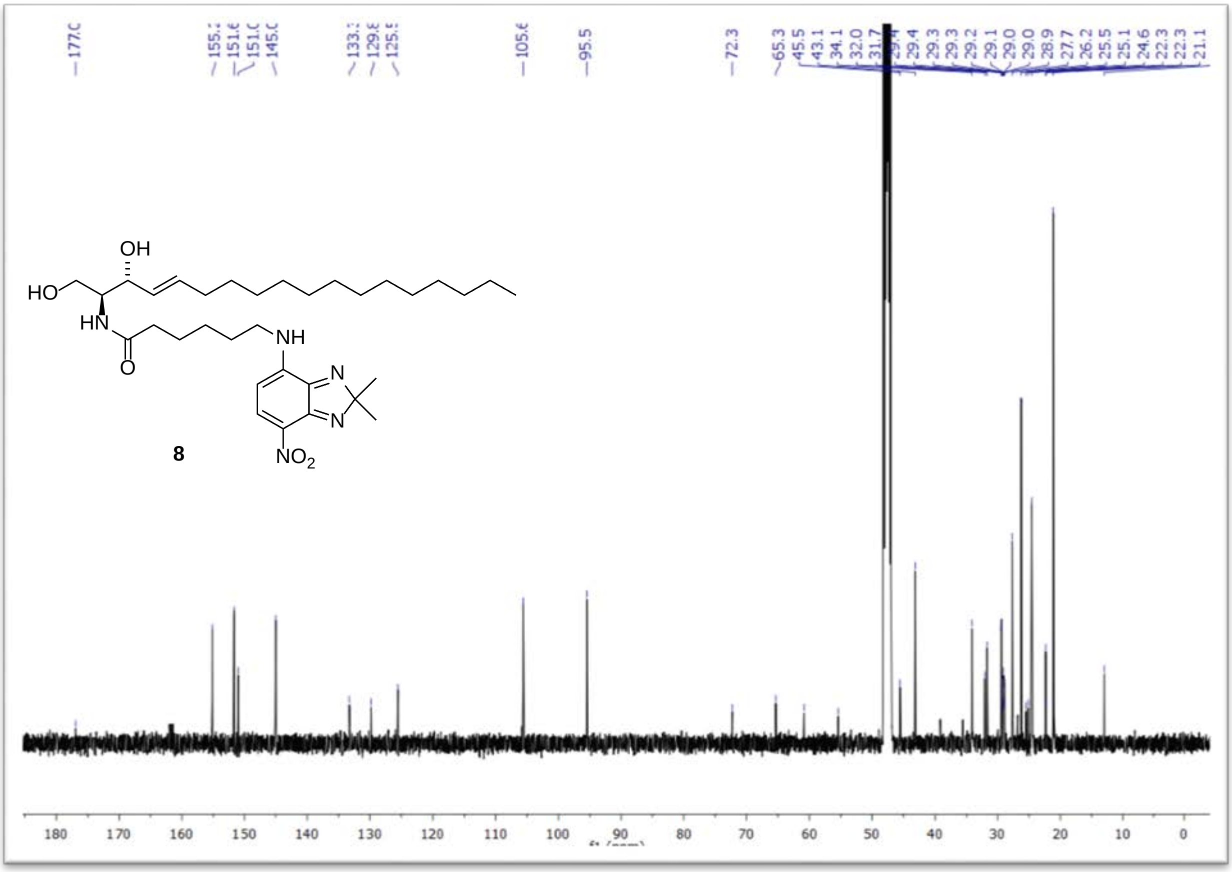

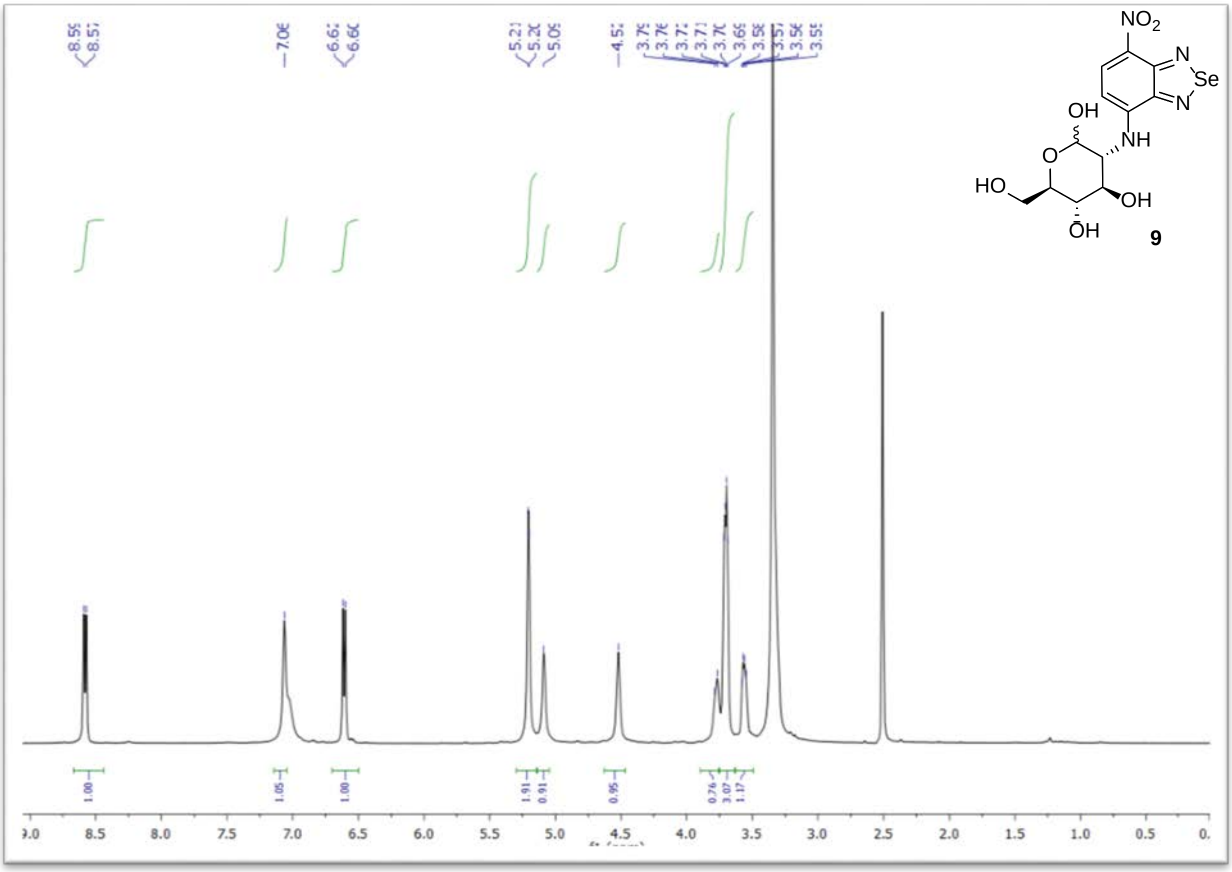
NO2
N
Se
N
OH
NH
O
HO
OH
OH
9
1.00
1.05
1.00
1.91
0.91
0.95
0.76
3.07
1.17

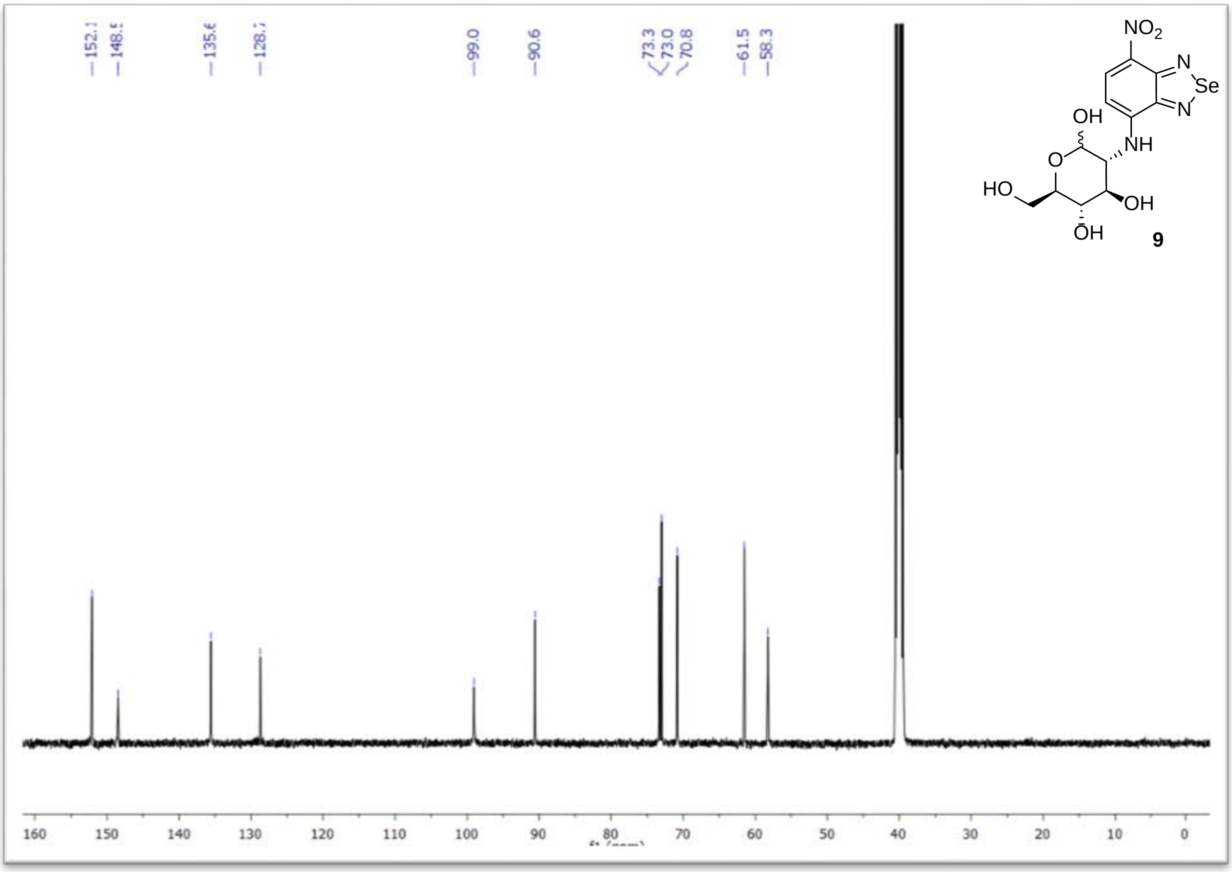
99.0
90.6
73.3
73.0
70.8
61.5
58.3
NO2
N
Se
N
OH
NH
O
HO
OH
OH
9

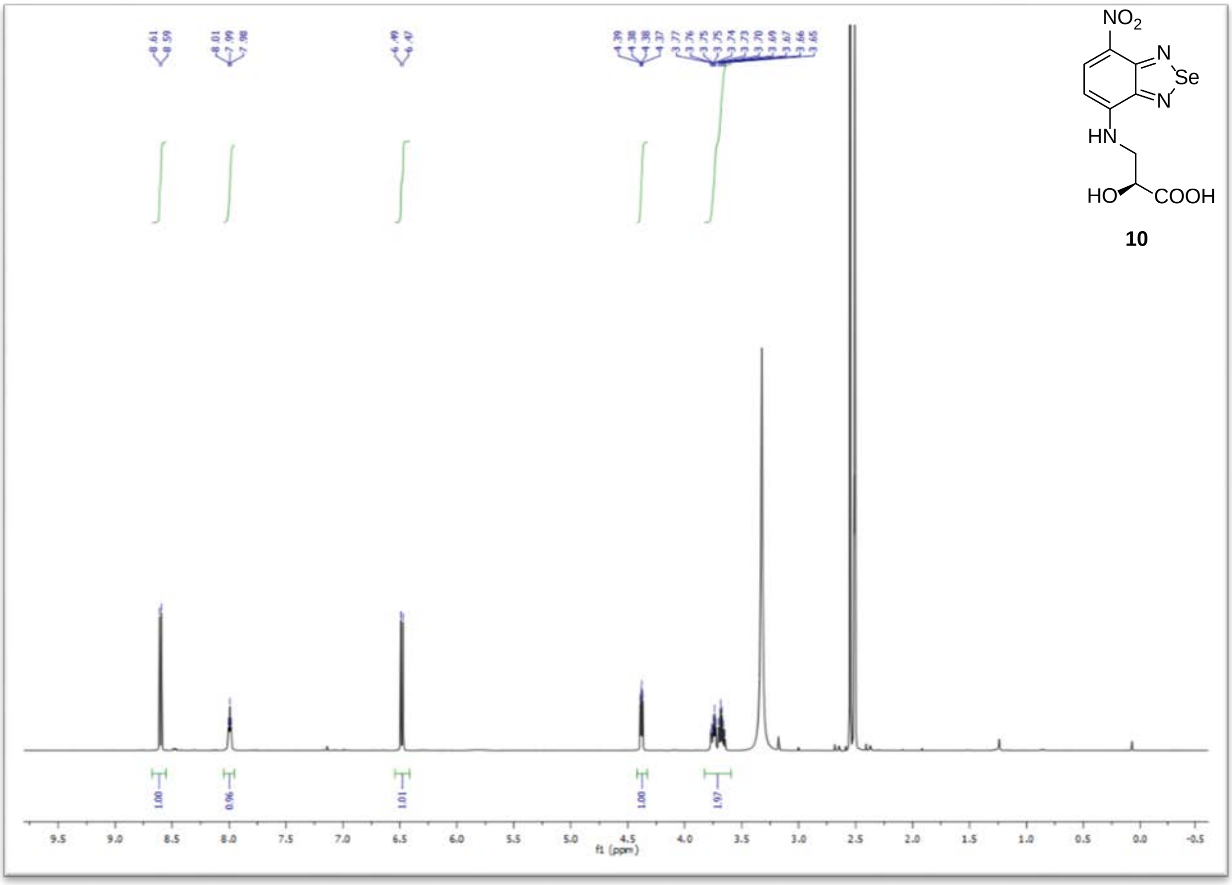
8.61
8.59
8.01
7.99
7.98
6.49
6.47
4.39
4.38
4.38
4.37
3.77
3.76
3.75
3.75
3.74
3.73
3.70
3.69
3.67
3.66
3.65
1.00
0.96
1.01
1.00
1.97
f1 (ppm)
NO2
N
Se
N
HN
HO
COOH
10

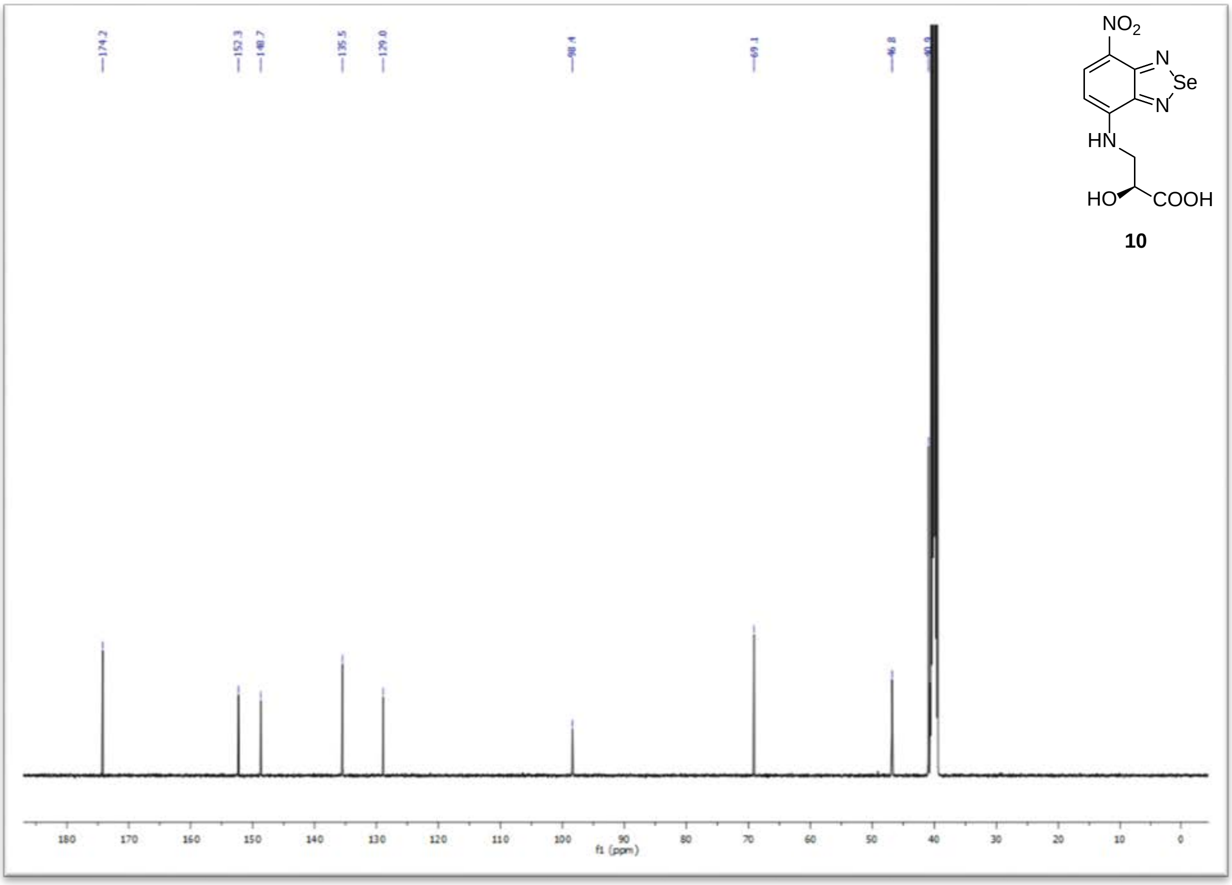
174.2
152.3
148.7
135.5
129.0
98.4
69.1
46.8
f1 (ppm)
NO2
N
Se
N
HN
HO
COOH
10

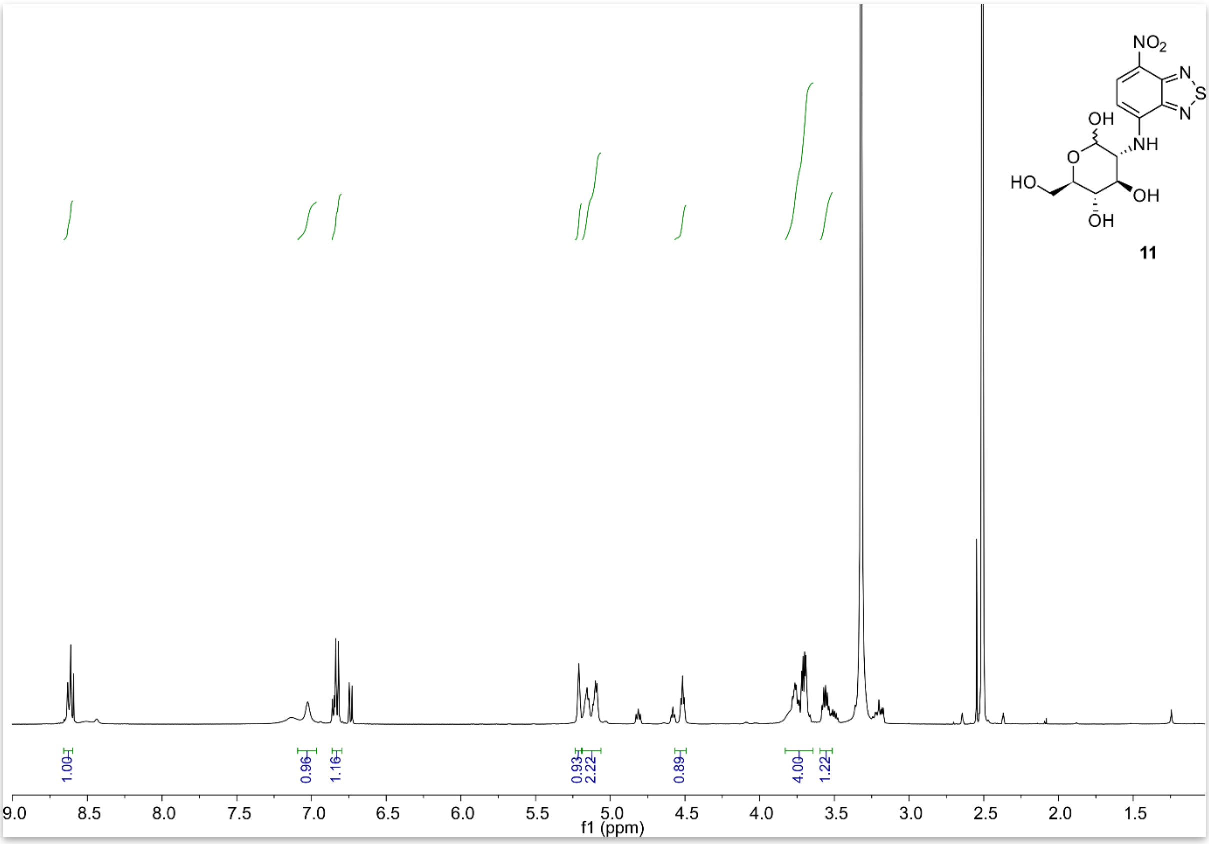

NO2
N
S
N
OH
NH
O
HO
OH
OH
11
1.00
0.96
1.16
0.93
2.22
0.89
4.00
1.22
9.0
8.5
8.0
7.5
7.0
6.5
6.0
5.5
5.0
4.5
4.0
3.5
3.0
2.5
2.0
1.5
f1 (ppm)

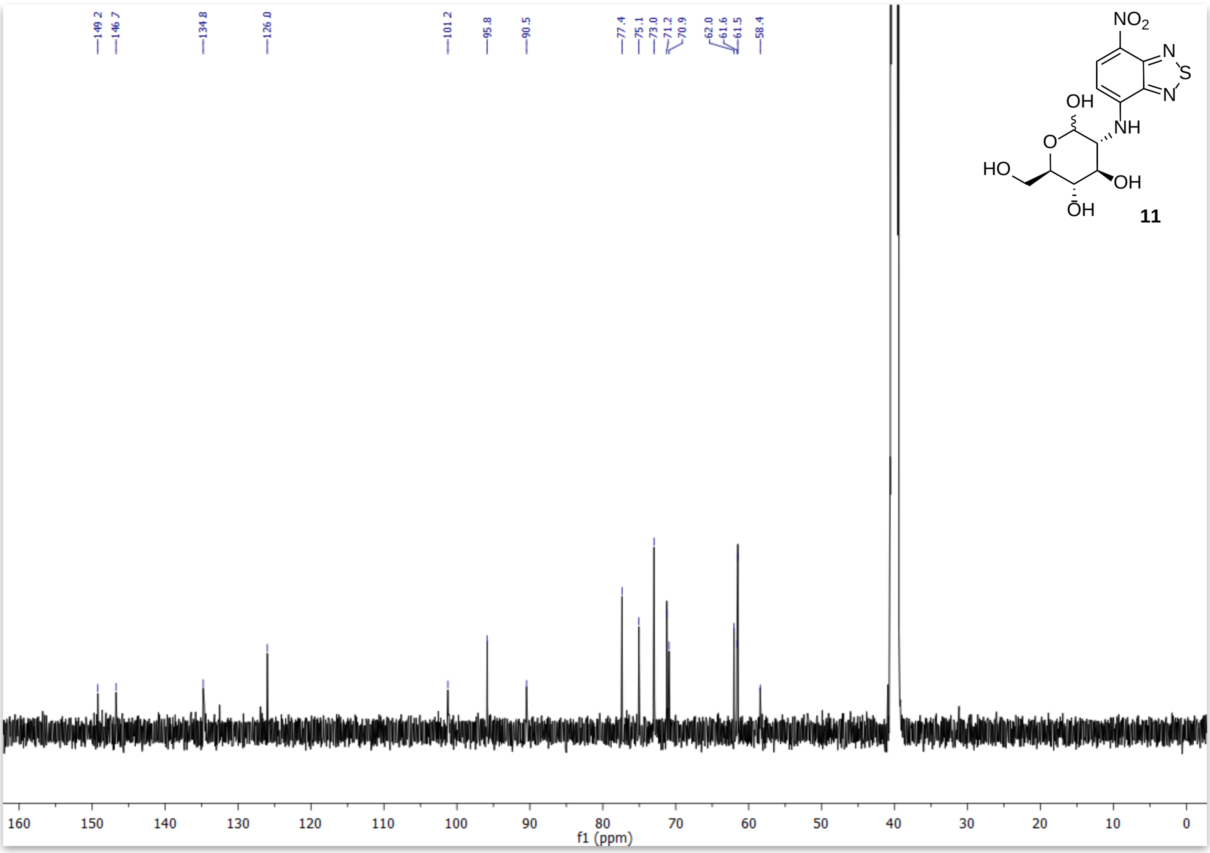

149.2
146.7
134.8
126.0
101.2
95.8
90.5
77.4
75.1
73.0
71.2
70.9
62.0
61.6
61.5
58.4
NO2
N
S
N
OH
NH
O
HO
OH
OH
11
160
150
140
130
120
110
100
90
80
70
60
50
40
30
20
10
0
f1 (ppm)

3. Supplementary Figures.

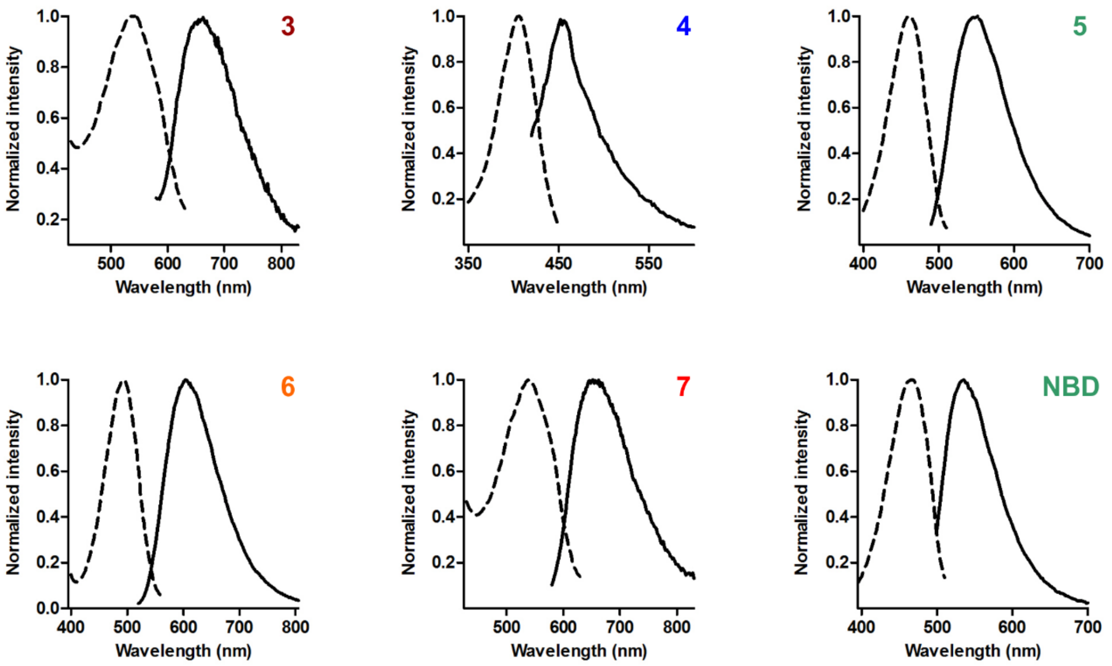

Figure S1. **Spectral characterization of SCOTfluors 3**-**7**. Absorbance (dashed line) and emission (solid line) spectra measured in EtOH. $\lambda_{exc.}$: 370 nm (**4**), 460 nm (**5**), 470 nm (**NBD**), 490 nm (**6**), 540 nm (**3** and **7**).

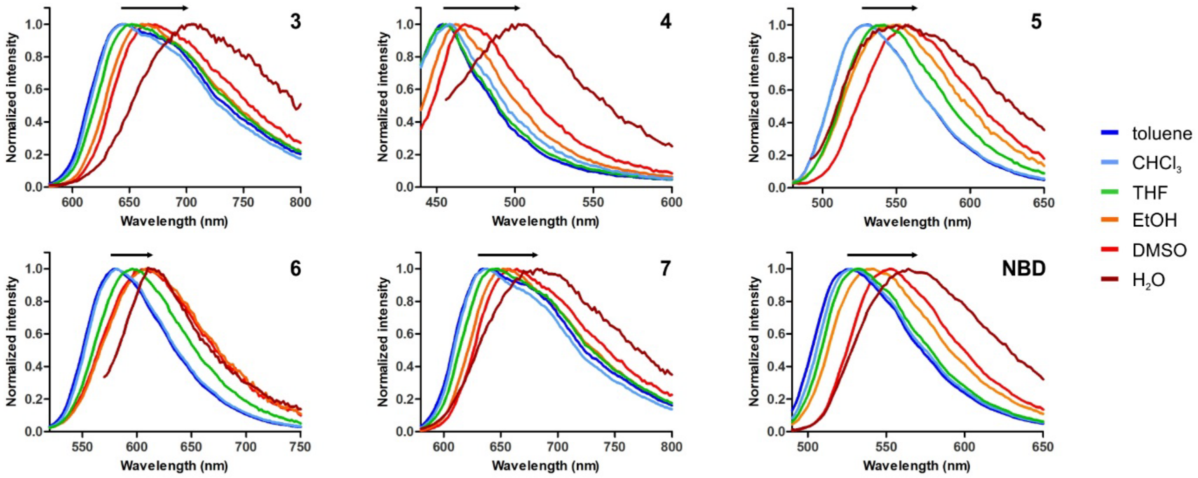

Figure S2. Solvatochromic behavior of SCOTfluors 3-7. Emission spectra for all compounds were acquired in solvents of different polarity and H-bonding capabilities. $\lambda_{exc.}$: 410 nm (**4**), 450 nm (**5**), 460 nm (**NBD**), 480 nm (**6**), 540 nm (**3** and **7**).

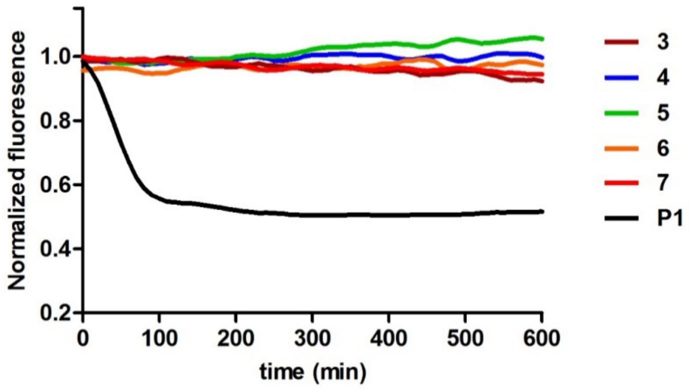

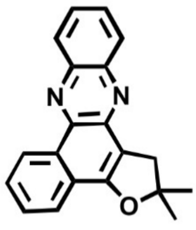

P1

	% stab.
**3**	91±2
**4**	99±2
**5**	99±7
**6**	95±3
**7**	93±1
**P1**	51±2

Figure S3. Photostability analysis of SCOTfluors 3-7. Longitudinal measurement of fluorescence emission of compounds **3**-**7** (100 μM) and the photo-unstable phenazine **P1** (100 μM) in PBS upon 10 h-irradiation at their maxima absorption wavelengths at 25˚C. The table summarizes the chemical stability values after 10 h as means ± s.d. (n = 3).

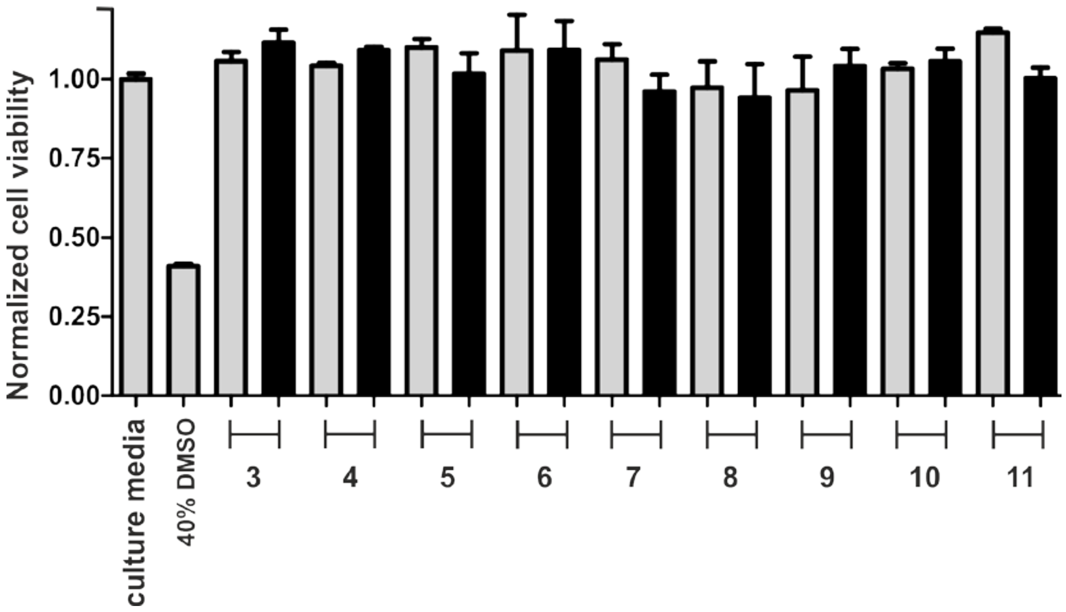

Figure S4. Cytotoxicity assays. HeLa cells were incubated with compounds **3-11** (100 μM, 1% DMSO, black bar; 75 μM, 1% DMSO, grey bar) for 1 h at 37˚C. Cell viabilities were normalized to those in cell culture media only under the same experimental conditions. A solution of 40% DMSO in culture media was used as a cytotoxic positive control. Values represented as means ± s.e.m. (n = 3).

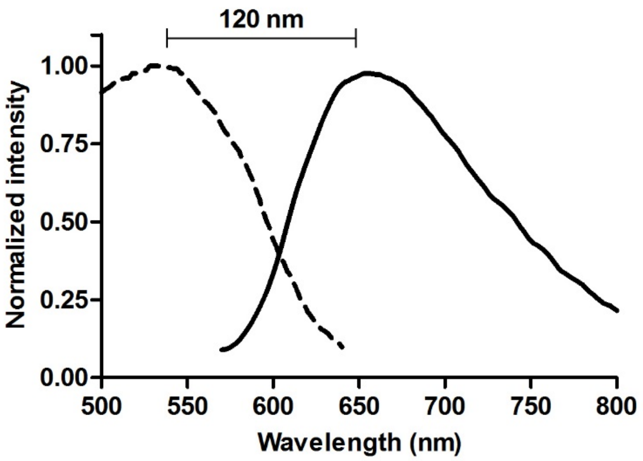

Figure S5. **Spectral characterization of the ceramide 8**. Absorbance (dashed line) and emission (solid line) spectra measured in EtOH. $\lambda_{exc.}$: 540 nm.

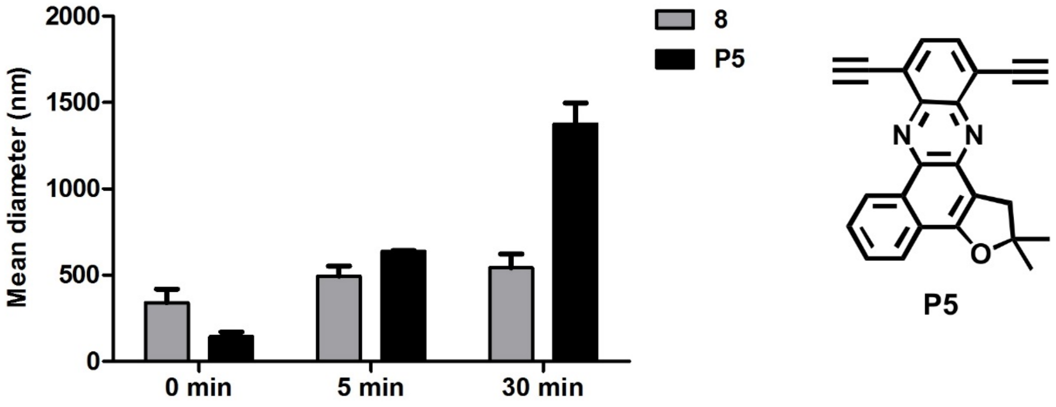

P5

Figure S6. Aggregation analysis. Time-course analysis of the mean aggregate size of the ceramide **8** and the aggregating phenazine **P5** (100 μM in PBS) determined by dynamic light scattering at r.t. Values represented as means ± s.e.m (n = 3).

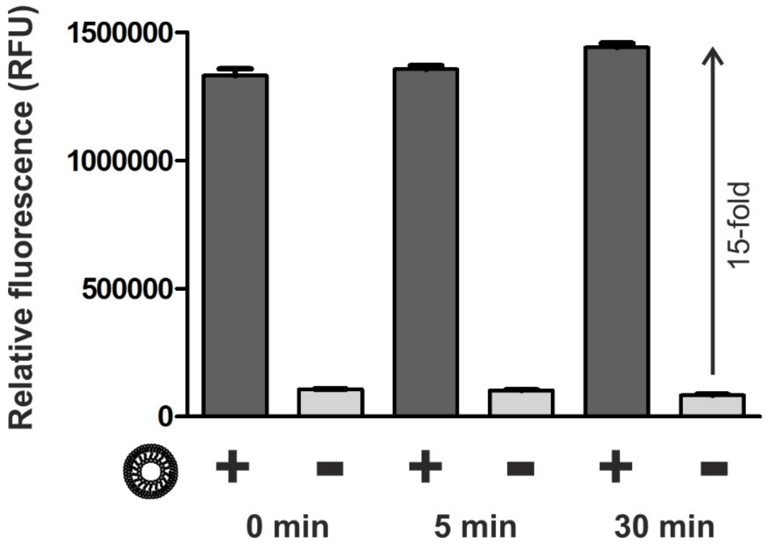

Figure S7. Liposome fluorescence activation of ceramide 8. Time-course analysis of the fluorescence intensity of ceramide **8** (100 μM) upon incubation with liposomes (dark grey) and PBS (light grey). Values represented as means ± s.e.m (n = 3).

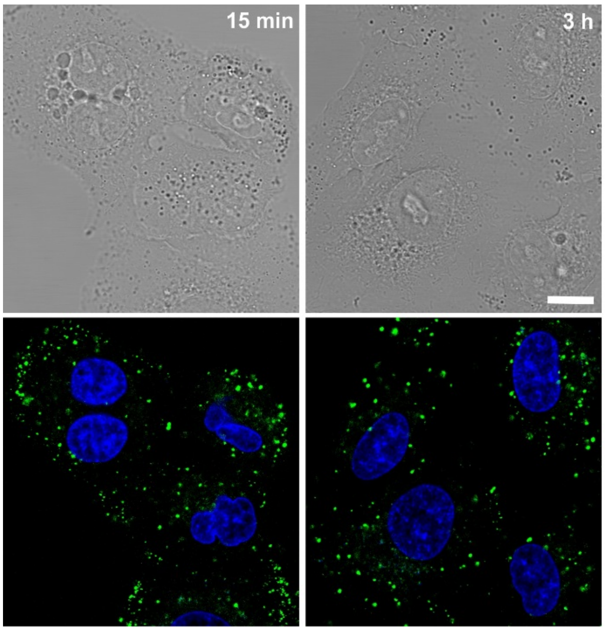

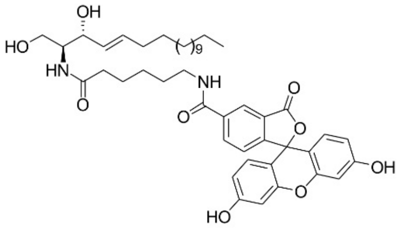

Cer-Fluor

Figure S8. Fluorescence microscopy of A549 cells with Cer-Fluor. Time-lapse confocal microscopy images of A549 cells treated with **Cer-Fluor** (1 μM, green) and Hoechst 33342 (nuclear counterstain, blue) show no differential localization of the ceramide analog after 15 min and 3 h. Scale bar: 15 μm.

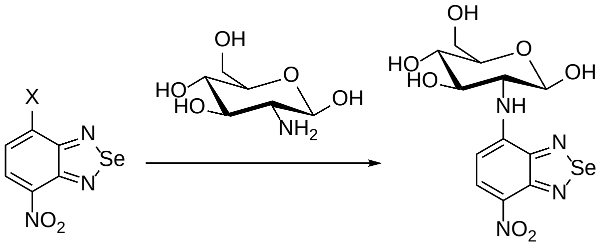

9

Starting material	Reaction conditions	Reaction time (h)	Recovery yield (%)
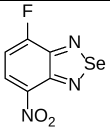	DMSO, r.t.	1.5	90
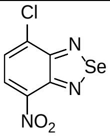	DMSO, 120˚C	20	7

Figure S9. Comparative reactivity of fluoride and chloride nitrobenzodiazoles with 2-amino-2-deoxy-D-glucose.

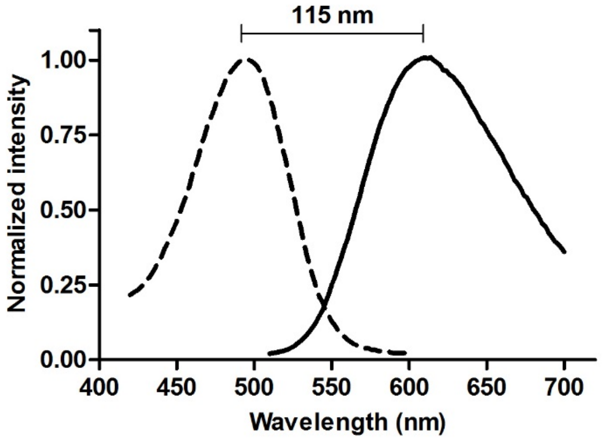

Figure S10. **Spectral characterization of glucose analog 9**. Absorbance (dashed line) and emission (solid line) spectra measured in EtOH. $\lambda_{exc.}$: 490 nm.

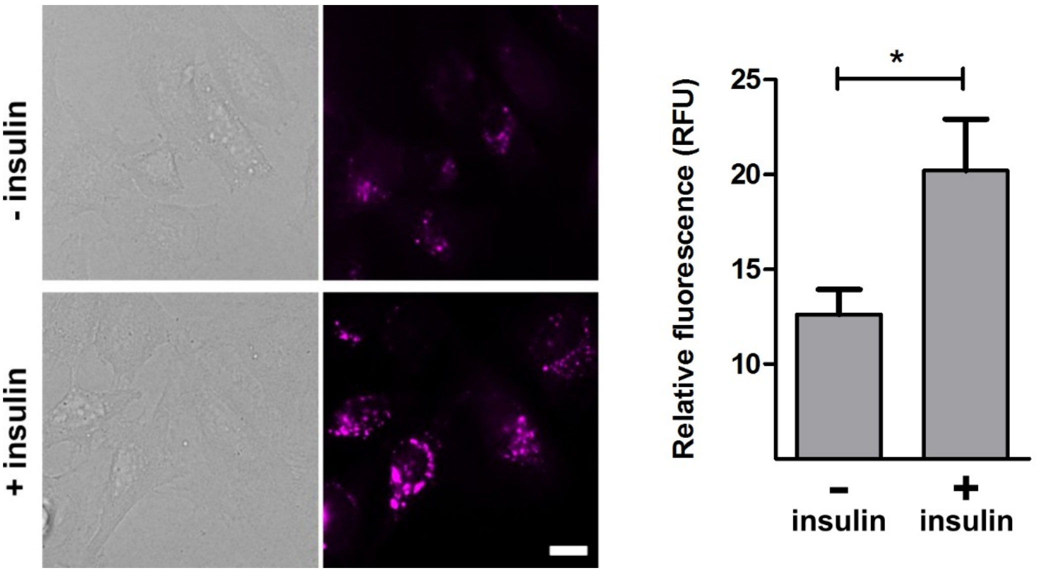

Figure S11. **Functional uptake of compound 9 in GLUT4-expressing cells after insulin treatment**. Confocal fluorescence microscope images and fluorescence quantification of HA-GLUT4 transfected cells after incubation or not with insulin (100 nM) and compound **9** (500 μM) for 1 h at 37˚C. $\lambda_{exc.}$: 488 nm. Values represented as means ± s.e.m; * for p value < 0.05 (n>3). Scale bar: 20 μm.

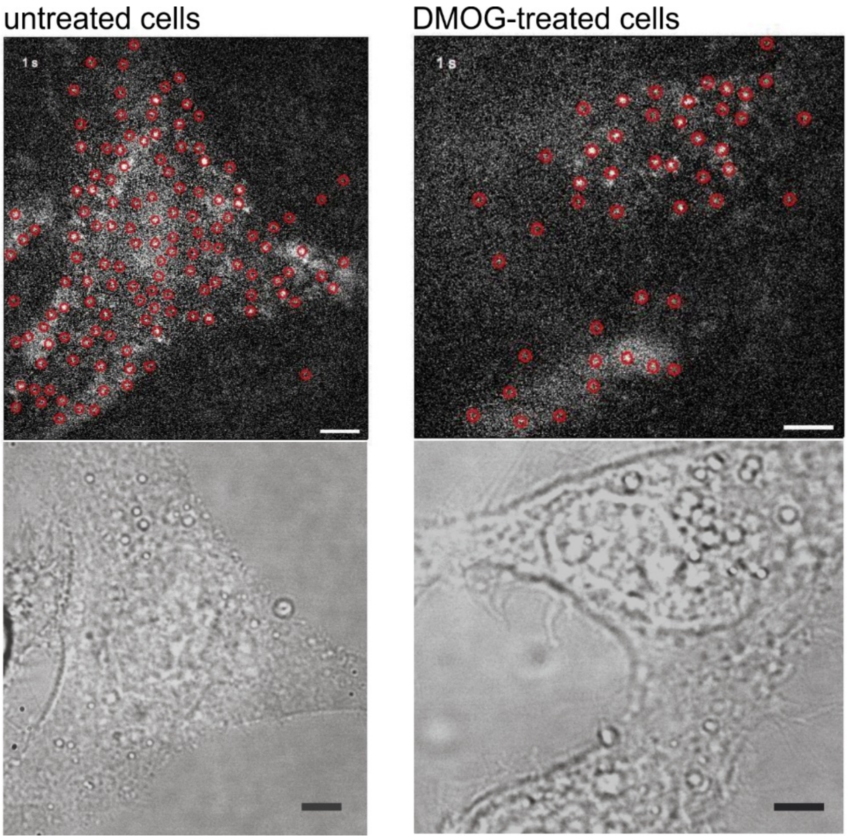

Figure S12. Super-resolution particle tracking in cells. Representative brightfield and fluorescence microscopy images corresponding to multiple tracked particles in untreated and DMOG-treated HeLa cells after incubation with compound **10** (100 μM). The corresponding movies are presented in Movie 3 (untreated) and Movie 4 (DMOG-treated). Scale bar: 5 μm.

Compound **10**

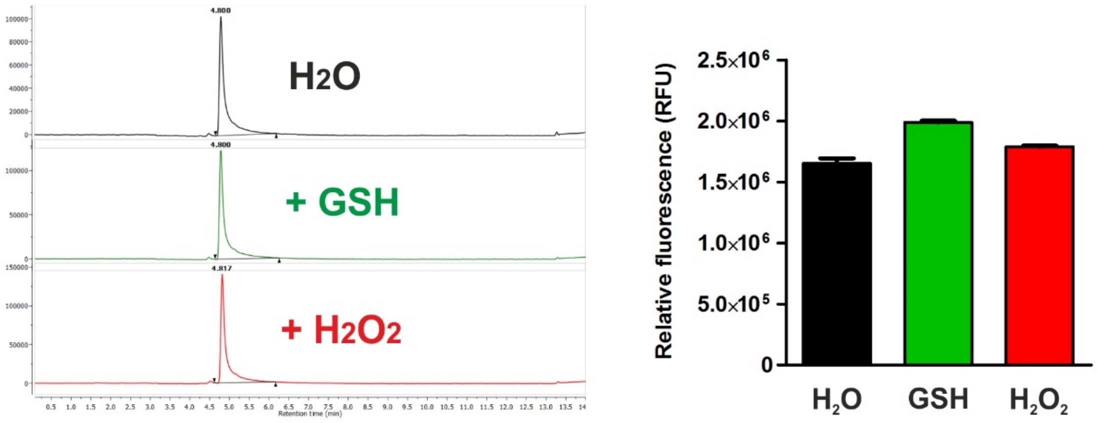

Compound **11**

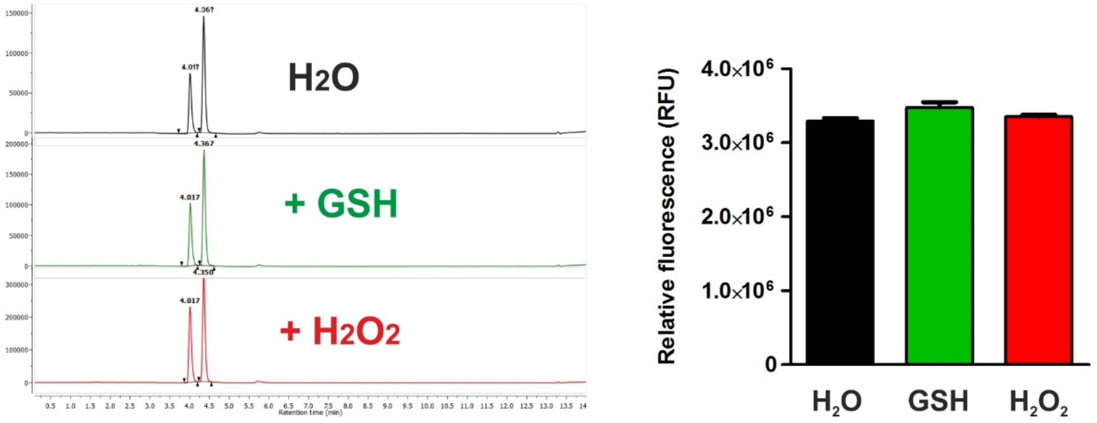

Figure S13. Stability assays of compounds 10 and 11. HPLC traces and fluorescence emission responses of compound **10** and compound **11** were obtained after incubation in water or buffers containing GSH (1 mM) and H_2O_2 (100 μM).

4. Biological Assays.

Cell culture and transfection. A549 and HeLa cells were obtained from LGC Standards and cultured in Dulbecco's modified Eagle's medium (DMEM) supplemented with 10% FBS, 100 U mL^{-1} penicillin and 0.1 mg mL^{-1} streptomycin. For transfection experiments, HeLa cells were transfected with pcDNA-PYP-GLUT4-HA-EGFP. Amplification of plasmid-DNA was performed by transformation of competent *E. coli* DH5a, followed by isolation of pcDNA-PYP-GLUT4-HA-EGFP using GeneJet Plasmid Miniprep kit (ThermoFisher) according to the manufacturer's instructions. Prior to transfection, pcDNA concentration was determined using NanoDrop. Transfection of HeLa cells was performed according to manufacturer's protocol with 1 µg pcDNA-PYP-GLUT4-HA-EGFP using Lipofectamine 3000. Non-transfected as well as transfected cells were maintained at 37 °C and 5% CO_2 throughout the imaging experiments.

Cell viability. Cell viability was determined using a TACSR MTT Cell Proliferation assay (Trevigen) following manufacturer's instructions. Briefly, cells were plated on 96-well plates reaching 90-95% confluence on the day of the experiment. Compounds were added to the cells at indicated concentrations and incubated at 37°C for 1 h. Then, cells were washed, treated according to the manufacturer's instructions and their absorbance values (570 nm) were measured in a Synergy HT spectrophotometer (Biotek). Cell viabilities were normalized to the proliferation of the cells in cell culture medium only.

Live-cell fluorescence microscopy. Confocal fluorescence microscopy was performed on a confocal Leica SP5 or a spinning disk microscope with excitation at

488 nm (ER Tracker, LysoTracker, EGFP & compound **9**), 561 nm (compound **8**) and 405 nm (Hoechst 33342). Prior to time-lapse imaging and imaging of HeLa cells, cells were incubated in Krebs-Ringer bicarbonate buffer (KRB) (129 mM NaCl, 4.7 mM KCl, 1.2 mM KH_2PO_4, 5 mM $NaHCO_3$, 10 mM HEPES; 2.5 mM $CaCl_2$, 1.2 mM $MgCl_2$ and 0.2% BSA, pH 7.4) at 37°C for 3h. When appropriate, HeLa cells were washed and imaged in KRB with 1 µM Hoechst 33342 at 37°C, 5%CO_2.

Isolation of human neutrophils and flow cytometry. *Ex vivo* experiments with fresh human peripheral blood from healthy donors were approved by the Accredited Medical Regional Ethics Committee (AMREC, reference number 15-HV-013). Peripheral blood polymorphonuclear cells from healthy volunteers were isolated as previously described. Briefly, citrated blood (3.8%) was centrifuged at r.t. at 350 g for 20 min, platelet-rich plasma was removed and separation from erythrocytes was achieved by Dextran sedimentation and fractionation of leukocytes using isotonic Percoll gradient. Polymorphonuclear leukocytes (>95% neutrophils) were harvested from the 70%-81% interfaces. Neutrophils were cultured in RPMI without glucose supplemented with penicillin/streptavidin. For experiments under hypoxic conditions, medium was pre-conditioned for at least 1 h at 1% O_2 and 37°C. Neutrophils were cultured in the absence or presence of 100 nM fMLP under hypoxic (1% $SpiO_2$, 37°C) or normoxic (21% $SpiO_2$, 37°C, 5% CO_2) conditions for 30 min. Compound **10** was added immediately to study uptake under different conditions, followed by washing with 20 mM HEPES, 140 mM NaCl and 0.1% BSA buffer. For the competition assays, the cells were pre-incubated for 1 h at 37°C, 5% CO_2 with 5 mM lactic acid. Flow cytometry data were analyzed using FlowJo software.

Zebrafish *in vivo* imaging. Anti-sense *glut2* morpholino (5′-ATGACCTGCAGAC AACAAGGACACC-3′)[1] was reconstituted to 1 mM in nuclease-free H_2O and injected at 4.2 ng/embryo into the one-cell stage. Embryos were maintained at 28.5 °C, according to standard protocols. Wild-type controls and *glut2* morphants were injected into the yolk sac at 28 hours post fertilization (hpf) with compound **6-NEt$_2$** (50 pmol) or compound **9** (50 pmol), then embedded in 1% low-melting point agarose for live imaging. Live imaging was performed 30 min post-injection on an inverted Leica SP8 confocal microscope using a HC PL APO CS2 10x/0.40 dry lens. Compounds **6-NEt$_2$** and **9** were excited at 488 nm and detected at 498-600 nm.

TIRF microscopy. Imaging was performed using a home-built, bespoke single-molecule total internal reflection fluorescence (TIRF) microscope. Compound **10** was excited at 515 nm. Collimated laser light at 515 nm (Cobolt Fandango-300 DPSS Laser System, Cobalt, Sweden) was aligned and directed parallel to the optical axis at the edge of a 1.49 NA TIRF Objective (CFI Apochromat TIRF 60×C Oil), mounted on an inverted Nikon TI2 microscope (Nikon, Japan). The microscope was fitted with a perfect focus system which auto-corrects the z-stage drift during imaging. Fluorescence collected by the same objective was separated from the returning TIR beam by a dichroic mirror DI02-R514 (Semrock) for 515 nm and was passed through appropriate filters [515 nm: BLP01-561R, FF01-607/36 (Semrock)]. Fluorescence was then passed through a 2.5× beam expander and recorded on an EMCCD camera (Delta Evolve 512, Photometrics) operating in frame transfer mode (EMGain = 11.5 e^-/ADU and 250 ADU/photon). Each pixel was 103 nm in length. For single-particle tracking, the images were recorded for 2,000 frames as 20 frames s^{-1}. The microscope was automated using the open source microscopy platform Micromanager.[2]

Image analysis (co-localization). Quantitative analysis of fluorescence images and determination of co-localization coefficients was performed using Image J using the plugin Coloc. Briefly, regions of interest in paired fluorescence channels (**8** vs LysoTracker, **8** vs ER Tracker) were defined, and then the plugin was launched. Co-localization coefficients were determined as the Mander's coefficient overlaps, with R values ranging between 0 and 1. R values around 1 indicate high co-localization between channels, and R values around 0 indicate very low co-localization between channels.

Single-particle tracking analysis. Single-particle tracking was performed using Trackpy for Python. Particles with a feature size of 15 pixels and a total brightness of 8,000 ADUs for each frame were selected. The detected particles were linked into tracks using a maximum displacement threshold of 10 pixels and a memory of 5 frames. Only those tracks that lasted for more than 5 frames were used in further analysis. For each of the tracks, the mean squared displacement was calculated. A custom written code in Igor Pro (Wavemetrics) was used to generate trajectories, movies, and for further analysis. For each of the mean squared displacement curves, the first five points were fit to a straight line to determine the instantaneous diffusion coefficient from the gradient. The log of these were then binned into histograms of diffusion coefficients.

Metabolic profiles of human cells. Human cell lines were plated in 384 well-plates as 20,000 cells/well and incubated in Krebs-Ringer bicarbonate buffer (KRB: 129 mM NaCl, 4.7 mM KCl, 1.2 mM KH_2PO_4, 5 mM $NaHCO_3$, 10 mM HEPES, 2.5 mM $CaCl_2$,

1.2 mM $MgCl_2$ and 0.2% BSA, pH 7.4) at 37 °C for 3 h, after which cells were incubated with compounds **8** (100 μM), **10** (25 μM), **11** (100 μM) for 1 h at 37 °C. Cells were washed and resuspended in KRB buffer. Fluorescence intensity measurements were taken on a Biotek Synergy H1 Hybrid Reader [compound **8** ($\lambda_{exc.}$: 560 nm; $\lambda_{em.}$: 650 nm), compound **10** ($\lambda_{exc.}$: 520 nm; $\lambda_{em.}$: 610 nm), compound **11** ($\lambda_{exc.}$: 430 nm; $\lambda_{em.}$: 550 nm)] and their emission values per cell were normalized against the fluorescence intensity of the corresponding solutions in KRB buffer.

5. Supplementary Movie Legends.

Movie Legend 1. Time-lapse TIRF microscopy movie corresponding to particle tracking depicted in Figure 4C in untreated HeLa cells after incubation with compound **10** (100 μM). Time is indicated as seconds and images were acquired every 50 ms and played at 30 frames per second. Scale bar: 1 µm.

Movie Legend 2. Time-lapse TIRF microscopy movie corresponding to particle tracking depicted in Figure 4C in DMOG-treated HeLa cells after incubation with compound **10** (100 μM). Time is indicated as seconds and images were acquired every 50 ms and played at 30 frames per second. Scale bar: 1 µm.

Movie Legend 3. Time-lapse TIRF microscopy movie corresponding to multiple particle tracking in untreated HeLa cells depicted in Figure S12 after incubation with compound **10** (100 μM). Time is indicated as seconds and images were acquired every 50 ms and played at 30 frames per second. Scale bar: 5 µm.

Movie Legend 4. Time-lapse TIRF microscopy movie corresponding to multiple particle tracking in DMOG-treated HeLa cells depicted in Figure S12 after incubation with compound **10** (100 μM). Time is indicated as seconds and images were acquired every 50 ms and played at 30 frames per second. Scale bar: 5 µm.

6. Supplementary References

1. Marin-Juez, R.; Rovira, M.; Crespo, D.; van der Vaart, M.; Spaink, H.P. & Planas, J. *J. Cereb. Blood Flow Metab.* **2015**, *35*, 74.

2. Edelstein, A.D.; Tsuchida, M.A.; Amodaj, N.; Pinkard, H.; Vale, R.D. & Stuurman, N. Advanced methods of microscope control using μManager software. *J. Biol. Methods* **2014**, *1*, e10.
